# Complete genome assembly of Hawai’i environmental nontuberculous mycobacteria reveals unexpected co-isolation with methylobacteria

**DOI:** 10.1371/journal.pone.0291072

**Published:** 2023-09-13

**Authors:** Jo Hendrix, L. Elaine Epperson, Eric I. Tong, Yvonne L. Chan, Nabeeh A. Hasan, Stephanie N. Dawrs, Grant J. Norton, Ravleen Virdi, James L. Crooks, Edward D. Chan, Jennifer R. Honda, Michael Strong

**Affiliations:** 1 Center for Genes, Environment, and Health, National Jewish Health, Denver, CO, United States of America; 2 Computational Bioscience Program, University of Colorado, Anschutz Medical Campus, Aurora, CO, United States of America; 3 ‘Iolani School, Honolulu, Hawai’i, United States of America; 4 Division of Biostatistics and Bioinformatics, National Jewish Health, Denver, CO, United States of America; 5 Department of Epidemiology, Colorado School of Public Health, Aurora, CO, United States of America; 6 Department of Medicine and Academic Affairs, National Jewish Health, Denver, CO, United States of America; 7 Division of Pulmonary Sciences and Critical Care Medicine, University of Colorado Anschutz Medical Campus, Aurora, CO, United States of America; 8 Department of Medicine, Rocky Mountain Regional Veterans Affairs Medical Center, Aurora, CO, United States of America; 9 Department of Cellular and Molecular Biology, School of Medicine, University of Texas Health Science Center at Tyler, Tyler, TX, United States of America; Lady Hardinge Medical College, INDIA

## Abstract

Nontuberculous mycobacteria (NTM) are ubiquitous environmental opportunistic pathogens that can cause chronic lung disease. Within the United States, Hawai’i has the highest incidence of NTM lung disease, though the precise reasons are yet to be fully elucidated. One possibility is the high prevalence of NTM in the Hawai’i environment acting as a potential reservoir for opportunistic NTM infections. Through our previous initiatives to collect and characterize NTM in Hawai’i, community scientists of Hawai’i have collected thousands of environmental samples for sequencing. Here, these community scientists were invited for the first time into a high school lab in O’ahu for a genomic sequencing workshop, where participants sequenced four of the collected isolate genomic samples using the Oxford Nanopore Technologies MinION sequencer. Participants generated high quality long read data that when combined with short read Illumina data yielded complete bacterial genomic assemblies suitable for in-depth analysis. The gene annotation analysis identified a suite of genes that might help NTM thrive in the Hawai’i environment. Further, we found evidence of co-occurring methylobacteria, revealed from the sequencing data, suggesting that in some cases methylobacteria and NTM may coexist in the same niche, challenging previously accepted paradigms. The sequencing efforts presented here generated novel insights regarding the potential survival strategies and microbial interactions of NTM in the geographic hot spot of Hawai’i. We highlight the contributions of community scientists and present an activity that can be reimplemented as a workshop or classroom activity by other research groups to engage their local communities.

## Introduction

Nontuberculous mycobacteria (NTM) are environmental opportunistic pathogens that can cause chronic lung disease in susceptible individuals, particularly those with preexisting lung conditions [1] like cystic fibrosis [2, 3]. NTM thrive in a variety of environments including soil and natural freshwater sources, but are especially well adapted to human-made plumbing systems [4].

Hawai’i has more NTM lung disease cases per capita than any other US state [5]. Our prior research of 62 households found that clinically important NTM species are prevalent in the Hawai’i environment [6] and may be the source of the high rate of NTM infections [7, 8]. To more broadly study NTM diversity, a Hawai’i-based community science network involving more than 200 local students and teachers from 11 different schools collected more than 2,800 environmental samples from across O’ahu, Kaua’i, Hawai’i Island, and Maui [9]. Results were shared at the Inaugural Hawai’i NTM Lung Disease Education and Research Conference [9] at ‘Iolani School in Honolulu in February 2020 in partnership with the ‘Āina-Informatics Network (AIN). AIN is an educational program pioneered by ‘Iolani School that was designed to bring genome sciences and bioethics into Hawai’i classrooms [10]. AIN leverages and applies the recent developments in long read genomic sequencing from the Oxford Nanopore Technologies (ONT) MinION, a portable, easy to use, and low-cost sequencer [11], to teach high school students how to perform genomic sequencing. We use this same sequencing technology in our research to characterize the genomes of diverse clinical and environmental NTM species [12], bringing MinION sequencing to the participants of the Hawai’i-based community science network.

Here, we describe an engaging hands-on sequencing activity that was held during the Inaugural Hawai’i NTM Lung Disease Education and Research Conference, where twenty-two community scientists worked together to sequence four bacterial isolate genomes with a MinION. The long reads were supplemented Illumina short reads from the same samples to construct hybrid genome assemblies. Subsequent analysis identified plasmids and genomic elements that revealed that some of the samples had methylobacteria, suggesting that methylobacteria were living in close proximity to NTM, and that they were isolated or co-isolated during the NTM culture selection process. Annotation of all identified genomes revealed the presence of genes implicated in metabolizing different carbon sources, regulating homeostasis of important metals, and for tolerating toxic compounds and antimicrobials. The results showcased here reveal novel insights into the environmental NTM that inhabit Hawai’i and the benefits of educational outreach activities.

## Materials and methods

### Volunteer participation

The Hawai’i NTM research project was approved by the Kaiser Hawai’i IRB (IRB# 00000402). Written consent to participate in the workshop was obtained from all adult participants. For minors under 18 years of age, written consent was obtained from parents or guardians.

### Environmental sample collection and processing

Bacterial samples were collected from the Hawai’i community science project following methods previously described [6]. Four of these samples were selected for whole genome sequencing based on their unexpected pink pigmentation, which is typical of methylobacteria but not NTM. Initial species identifications were determined through single locus PCR amplification and Sanger sequencing of the *rpoB* gene, which resulted in a mycobacterial identification for each sample. The samples were named HI01-04.

HI01 was isolated from a kitchen sink on the island of Hawai’i and was identified as *Mycobacterium abscessus*. HI02 was isolated from a garden hose on the island of O’ahu and was identified as *Mycobacterium porcinum*. HI03 was isolated from a beach showerhead on the island of O’ahu and was identified as *Mycobacterium intracellulare*. HI04 was isolated from a household showerhead on the island of Hawai’i and was identified as *Mycobacterium avium* (Table 1).

For textual clarity, the genus name *Methylobacterium* is abbreviated as *Me*. to distinguish methylobacterial species from mycobacterial species (abbreviated as *M*.).

**Table 1:** Origin and *rpoB* identification of workshop isolates.

*ID*	*Island*	*Sample location description*	*Taxa ID*	*Colony color*
*HI01*	Hawai’i	Kitchen sink	*M*. *abscessus*	Pink
*HI02*	O’ahu	Garden hose	*M*. *porcinum*	Buff-white
*HI03*	O’ahu	Beach showerhead	*M*. *intracellulare*	Pink
*HI04*	Hawai’i	Showerhead	*M*. *avium*	Pink

### Bacterial culture and genomic DNA extraction

One mL of bacterial sample glycerol stock was added to 50mL of 7H9 broth. Cultures were incubated on a shaker at the sample’s original culture temperature, either 30°C or 37°C, until turbid (7–11 days). Once turbid, the cultures were centrifuged for 10min at 5,000 rpm to pellet the bacteria. The supernatants were discarded and the bacterial pellets were resuspended in 1mL of sterile 1xPBS. The resuspended samples were transferred into new sterile 1.5mL tubes, and centrifuged for 2min at 13,000xg. The supernatants were discarded and bacterial pellets were stored at 4°C until same-day gDNA extraction was initiated.

Before the workshop, bacterial culture stocks were grown (S2 File) and high molecular weight DNA was extracted according to Epperson et al. [13]. This protocol uses minimal biohazardous reagents and is compatible with student use. High concentration intact DNA samples were eluted, quantified, and stored at 4°C or room temperature until sequencing.

### Illumina sequencing

The same pools of DNA used for ONT sequencing were also packaged into sequencing libraries using the DNA Prep kit from Illumina (P/N 20018705) and sequenced on a MiSeq using 2X300bp paired end chemistry. Sequence reads were trimmed using Skewer v0.2.2 [14] during which the adapter sequence 5′-CTGTCTCTTATACACATCT-3′ was removed, read ends were trimmed for a minimum quality of 20, and reads were filtered for a minimum length of 40 bases after trimming.

### ONT sequencing

Workshop participants, which included high school students, college students, teachers, and mentors, were briefed on the sample origins and the laboratory techniques used to extract high molecular weight gDNA (S1 File). These modules were adapted from AIN’s extensive curriculum centered around MinION sequencing for high school students, providing the instructional component for this workshop [10]. In groups of 5 to 6 people, participants prepped DNA libraries for sequencing using the Rapid Barcoding Kit (SQK-RBK004) from ONT, where 2.5μl of fragmentation mix was added to each 400ng sample to cleave DNA and attach barcoded adapters. Each student group received their own unique barcode. Barcoded samples were pooled in pairs: HI01 was pooled with HI02, and HI03 was pooled with HI04. Sequencing adapters were attached by adding 1μl of rapid adapter (RAP) to 10μl of barcoded DNA. After checking the number of active pores and priming the flow cell, the library was prepared for sequencing by mixing the sequencing buffer (SQB) and loading beads (LB).

The pooled libraries were loaded onto two separate MinION flow cells (FLO-MIN106D R9.4.1). Flow cell 1 started with 1,271 active pores and was loaded with samples HI01 and HI02. Flow cell 2 started with 1,352 active pores and was loaded with samples HI03 and HI04. Sequencing was performed for 22 hours and 27 minutes. By the end of the sequencing round, flow cell 1 had 433 active pores and flow cell 2 had 760 active pores. Raw ONT reads were subjected to high accuracy basecalling and demultiplexing using Guppy v3.4.5 [15].

### Assembly and annotation

Following the workshop, the ONT and Illumina reads were assembled using Unicycler v0.4.4 [16] to construct hybrid genome assemblies. Assemblies were annotated with Prokka v1.13.3 [17]. Roprokka (https://github.com/jrhendrix/Roprokka) was used to count the number of *dnaA* genes and to determine the species of origin for *16S* and *rpoB* gene sequences.

We grouped the contigs by likely genus of origin based on the presence of a species-identifying gene or connections to another contig with such a gene. Unconnected contigs less than 200,000bp in length were aligned to the BLAST nr database and sorted by sequence homology. Average Nucleotide Identity (ANI) analysis of the entire genome relative to known bacterial species was performed by GenBank.

### Microbiological screening of mixed genome samples

Following sequencing, samples that indicated possible methylobacterial genomes were restreaked onto Middlebrook 7H10 agar plates supplemented with OADC (oleic acid, albumin, dextrose, and catalase). Plates were incubated at 30°C for 13 days at which point two samples displayed two distinct morphologies. Per plate, one colony was picked to represent each morphology. Colonies were smeared onto duplicate microscope slides then heat fixed. Sample slides were stained for acid fast bacilli (AFB) using Kinyoun cold method (Hardy Diagnostics) or Gram stained (Fluka Analytical) as per manufacturer’s instruction. Stained samples were visualized by light microscopy at 1000x magnification (SeBa, Laxco).

To determine whether distinct colony morphologies could be separated, each colony morphology was also inoculated into Middlebrook 7H9 broth. Liquid cultures were incubated at 30°C until turbid. Once turbid, 10μl was streaked onto Middlebrook 7H10-OADC agar supplemented with 2% malachite green. Plates were incubated at 30°C.

## Results and discussion

### ONT sequencing

**Fig 1:**
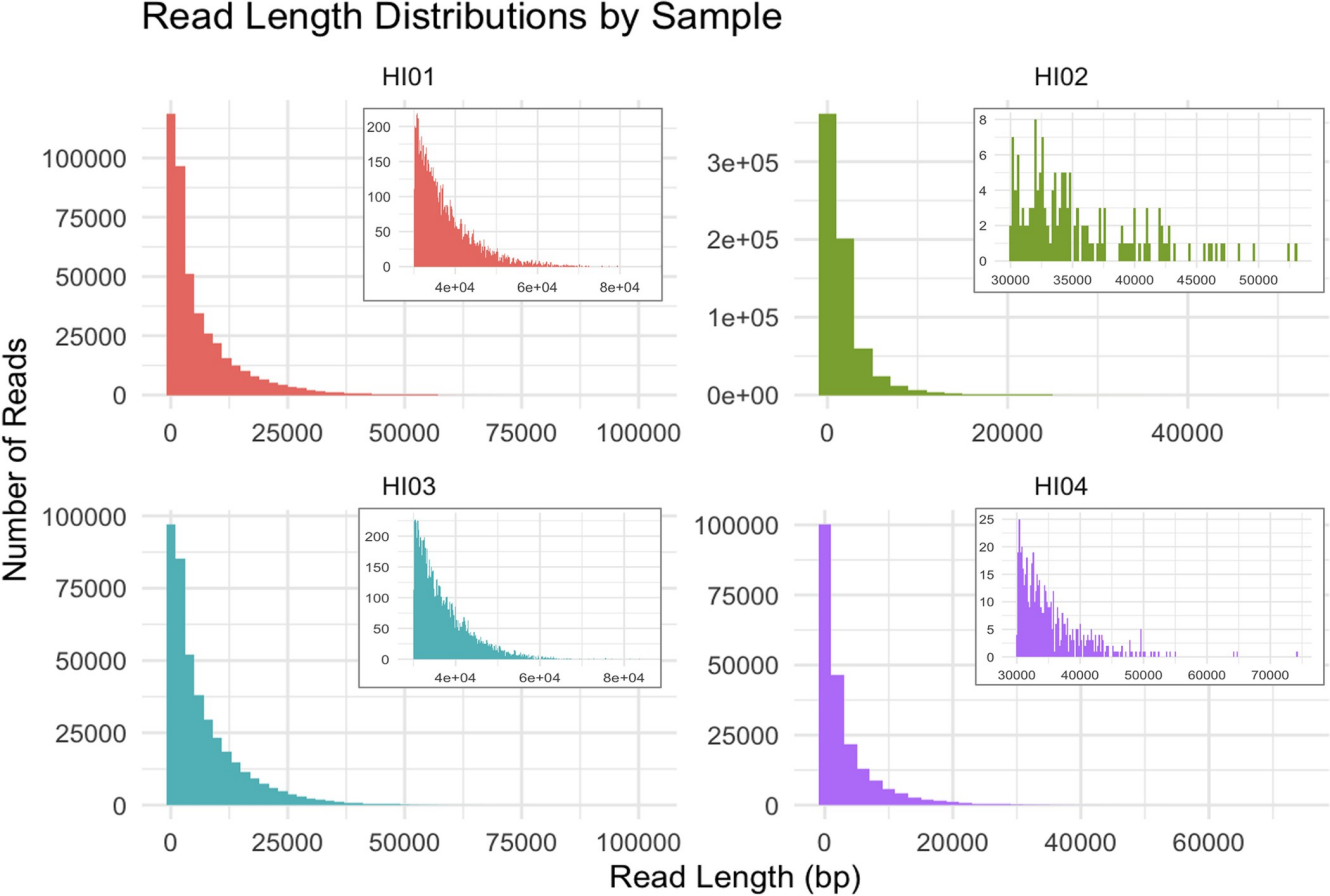
ONT read length distribution. The length of ONT reads followed a pattern of exponential decay with fewer longer reads. For ease of viewing, the distributions of read lengths greater than or equal to 30,000 are shown as insets.

Two MinION device sequencing runs were performed, generating 7.4Gb of data on flow cell 1 and 7.8Gb of data on flow cell 2. Read length distributions can be seen in Fig 1. The number of sequences per sample varied from just over 200,000 to nearly 700,000 reads with 95.6–98.8% reads having an average quality score greater than 14.0. Sample HI04 had the fewest reads and the lowest ONT read coverage of 46x while the other three samples had ONT read coverages greater than 150x.

### Assembly and annotation

**Fig 2:**
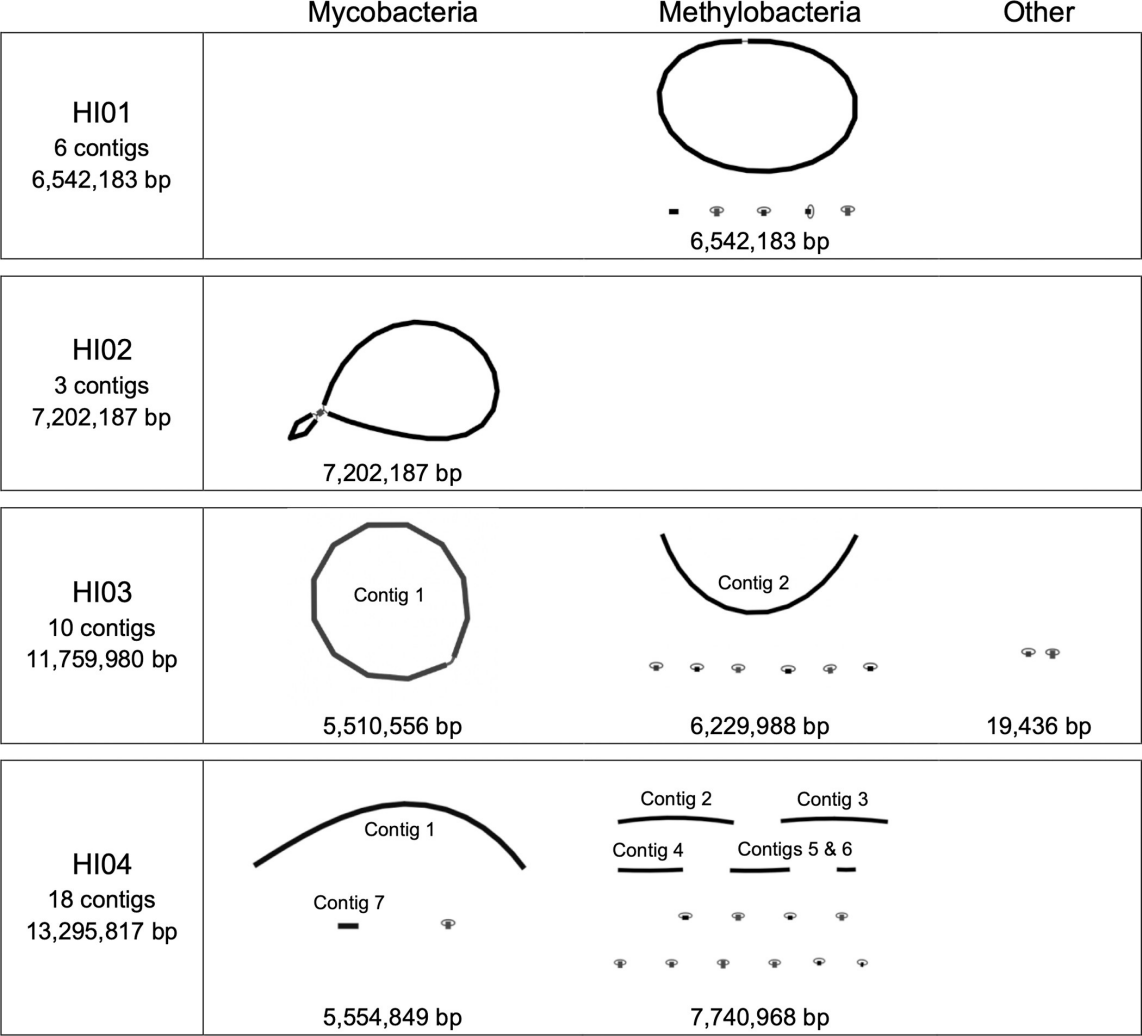
Assembly graphs of samples HI01-04 where contigs are partitioned by genus. Thicker lines represent DNA contigs (segments pieced together with high confidence.) Thinner lines between contigs indicate that the assembler found some evidence that the contigs are contiguous. Circularized plasmids are indicated by a thick contig line with both ends connected by a thinner line. Contigs identified as being from mycobacterial origin are on the left, contigs identified as methylobacteria are in the middle, and contigs that either aligned to a different genus or could not be assigned are shown on the right. For each genus grouping, the total DNA content is recorded under the assembly graph. Features discussed in the text are labeled. Contig sizes are not drawn to scale.

Genomic sample HI01 was assembled into six contigs that totaled 6,542,183 bp. The main chromosomal unit consisted of a single circularized contig that contained five *16S* and one *rpoB* annotation from *Methylobacterium* (*Me*.) *populi*. Five independent contigs were identified: four circularized with sequence similarity to methylobacterial plasmids and one uncircularized sequence with slight sequence similarity to existing methylobacterial genomes (Fig 2, top panel). GenBank ANI analysis identified this genome as *Methylobacterium* (*Me*.*) rhodesianum*. HI01 contained genes involved in the import of ammonia (*amt*, *amtB*), the degradation of nicotinic acid (*nicA*, *nicB*), and the homeostasis of iron (*iscA*), magnesium (*corA*, *mgtA*), and divalent cations (*mntH*). Various genes suggest decreased sensitivity to arsenic (*arc3*, *arsB*, *arsC*), cadmium (*cadA*), copper (*copA*), mercury (*merR*, *merR1*), lithium (*nhaK*), zinc (*zntA*), and general organic hydroperoxide (*ohrB*). Some genes also conferred resistance to the antibiotics beta-lactams (*ampC*), bicyclomycin (*bcr*), fosmidomycin (*fsr*), and multi-drug families (*sugE*) (Table 2).

**Table 2:** Presence of select annotations per genome.

Function	Gene	HI01-Me	HI03-Me	HI04-Me	HI02-NTM	HI03-NTM	HI04-NTM	Citation
Metabolism								
Ammonium import	*amt*	✓			✓	✓	✓	[18]
	*amtB*	✓	✓	✓				[18]
Caffeine breakdown	*cdhA*		✓	✓	✓			[19]
	*cdhB*		✓		✓			[19]
	*cdhC*		✓	✓	✓			[19]
Nicotinic acid breakdown	*nicA*	✓	✓	✓				[20]
	*nicB*	✓	✓	✓				[20]
Metal Homeostasis								
Iron transport	*irtA*				✓	✓	✓	[21]
	*iscA*	✓	✓	✓				[22]
Magnesium transport	*corA*	✓	✓	✓	✓	✓	✓	[23, 24]
	*mgtA*	✓	✓	✓	✓	✓	✓	[25]
Divalent cation transport	*mntH*	✓	✓	✓	✓	✓	✓	[26, 27]
Toxic Metal Tolerance								
Arsenic resistance	*acr3*	✓	✓		✓		✓	[28]
	*arsB*	✓	✓	✓	✓	✓	✓	[29]
	*arsC*	✓	✓	✓	✓	✓	✓	[30]
Cadmium transport	*cadA*	✓						[31]
Copper tolerance	*copA*	✓	✓	✓	✓	✓		[32–34]
	*mctB*				✓	✓	✓	[35]
Heavy metal tolerance	*atm1*	✓	✓	✓				[36]
Lithium transport	*nhaK*	✓	✓	✓				[37]
Mercury resistance	*merA*	✓	✓					[38]
	*merR*	✓	✓			✓		[39]
	*merR1*	✓	✓		✓		✓	[39]
Organic hydroperoxide tolerance	*ohrB*	✓	✓	✓	✓			[40]
Zinc tolerance	*zntA*	✓	✓	✓				[41]
Antibiotic Resistance								
β-lactam resistance	*ampC*	✓	✓		✓			[42]
	*bla*			✓				[43]
Bicyclomycin resistance	*bcr*	✓	✓	✓	✓			[44]
Daunorubicin, doxorubicin resistance	*drrA*			✓	✓	✓	✓	[45]
	*drrB*				✓	✓	✓	[45–47]
Isoniazid resistance	*iniA*				✓			[48]
Fosmidomycin resistance	*fsr*	✓	✓	✓				[49]
Multidrug resistance	*mmr*					✓	✓	[50]
	*stp*			✓	✓	✓	✓	[51]
	*sugE*	✓	✓	✓	✓	✓	✓	[52, 53]

Genomic sample HI02 was 7,202,187 bp with evidence of all three contigs being part of a single chromosomal unit. Two *16S* and one *rpoB* gene were identified as *Mycobacterium* (*M*.) *porcinum* (Fig 2, second panel). GenBank ANI analysis also identified this genome as *M*. *porcinum*. Genome annotation of HI02 revealed genes involved in nutrient acquisition from ammonia (*amt*), caffeine (*cdhA*, *cdhB*, *cdhC*), and homeostasis of iron (*irtA*), magnesium (*corA*, *mgtA*), and divalent cations (*mntH*). The genome also contained genes that lower susceptibility to arsenic (*acr3*, *arsB*, *arsC*), cadmium (*cadI*), copper (*copA*, *mctB*), mercury (*merR1*), and organic hydroperoxide (*ohrB*). The annotations included genes that confer resistance to beta-lactams (*ampC*), bicyclomycin (*bcr*), daunorubicin and doxorubicin (*drrA*), ethambutol and isoniazid (*iniA*), and multi-drug families (*sugE*) (Table 2).

Genomic sample HI03 was assembled into ten contigs that totaled 11,759,980 bp. Contig 1 contained a *16S* and *rpoB* annotation identified as *Mycobacterium* (*M*.) *intracellulare*. Contig 2 contained five *16S* genes and one *rpoB* gene from *Me*. *populi*. Copies of the *dnaA* gene were present on contigs 1 and 2. Of the eight unconnected small contigs, there were six methylobacterial plasmids, one *Brucella anthropi* plasmid, and one unidentified plasmid (Fig 2, third panel). The contigs associated with NTM and methylobacterial DNA were separated into assemblies HI03-NTM and HI03-Me, respectively. GenBank ANI analysis identified these genomes as *Me*. *populi* (HI03-Me) and *M*. *intracellulare* (HI03-NTM). HI03-NTM contained genes involved in ammonia transport (*amt*), iron homeostasis (*irtA*) along with improved tolerance of copper (*mctB*), and zinc (*zntA*) and resistance to daunorubicin and doxorubicin (*drrAB*), and multi-drug families (*mmr*, *stp*). The DNA associated with *Me*. *populi* in HI03-Me included genes to extract nutrients from ammonia (*amtB*), caffeine (*cdhA*B*C*), and nicotinic acid (*nicAB*), maintain proper iron levels (*iscA*), and improve tolerance of arsenic (*acr3*), organic hydroperoxide (*ohrB*), lithium (*nhaK*), mercury (*merR1*), and zinc (*zntA*) along with resistance to drugs beta-lactams (*ampC*), bicyclomycin (*bcr*), and fosmidomycin (*fsr*). Both genomes contained genes for maintaining homeostasis of magnesium (*corA*, *mgtA*, *mntH*), and increasing tolerance to arsenic (*arsBC*), copper (*copA*), mercury (*merR*), and multi-drug families (*sugE*) (Table 2).

Genomic sample HI04 contained 13,295,817 bp with 18 contigs. Contig 1 consisted of 5.5Mb with one of each *dnaA*, *16S*, and *rpoB* annotations from *Mycobacterium* (*M*.) *avium*. Contigs 2, 3, and 4 included two, six, and three *16S* genes, respectively, all from *Methylobacterium* (*Me*.) *aquaticum*. Contig 4 also contained a *dnaA* and *rpoB* gene from *Me*. *aquaticum*. Contigs 5 and 6 had high sequence similarity to methylobacterial chromosomes while contig 7 had highest similarity to mycobacterial chromosomes. Of the small-unconnected contigs there were ten methylobacterial plasmids and one plasmid identical to p18K in *M*. *avium* (Fig 2, bottom panel). The contigs associated with NTM and methylobacterial DNA were separated into assemblies HI04-NTM and HI04-Me, respectively. GenBank ANI analysis identified these genomes as *Methylobacterium ajmalii* (HI04-Me) and *M*. *avium* (HI04-NTM). Contigs in assembly HI04-NTM contained genes for metabolizing ammonia (*amt*) maintaining iron homeostasis (*irtA*), increasing tolerance to copper (*mctB*) and mercury (*merR1*), and conferring resistance to a multi-drug family (*mmr*). The HI04-Me assembly included genes for extrapolating nutrients from ammonia (*amtB*), caffeine (*cdhAC*), and nicotinic acid (*nicAB*), maintaining iron homeostasis (*iscA*), improving tolerance to chromate (*chrA1*, *srpC*), copper (*copA*), organic hydroperoxide (*ohrB*), lithium (nhaK), and zinc (*zntA*), and conferring resistance to beta-lactams (*bla*), bicyclomycin (*bcr*), and fosmidomycin (*fsr*). Both genomes contained genes to maintain magnesium homeostasis (*corA*, *mgtA*, *mntH*), increase tolerance of arsenic (*acr3*, *arsBC*) and resistance to daunorubicin and doxorubicin (*drrA*) and multi-drug families (*stp*, *sugE*) (Table 2).

**Fig 3:**
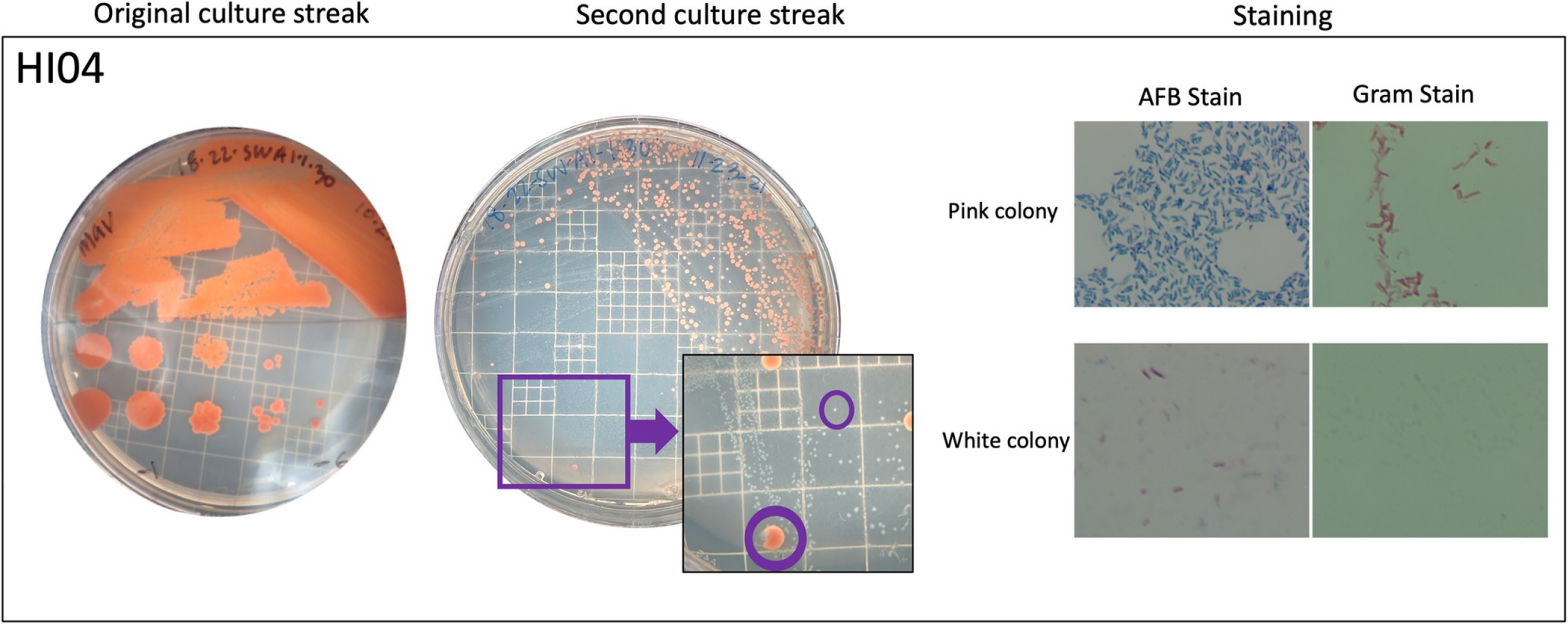
Replating of HI04 to show NTM and methylobacteria separation. Original culture streak of HI04 showing uniform pink colonies (left). HI04 was streaked a second time on 7H10-OADC plates and incubated for 13 days at 30°C, circled in purple are the two colony morphologies that appeared (center). The distinct pink and white colonies of HI04 were AFB and Gram stained and visualized by light microscopy at a total magnification of 1000X (right).

Samples HI01, HI03, and HI04 contained methylobacterial genomes and were subjected to additional microbiological culturing to determine if the cultures were true single-colony isolates as originally thought. When grown on 7H10-OADC plates, each sample produced pink medium-sized colonies after three days of incubation. By day 13, HI03 and HI04 produced a second morphotype that was small and white, typical of MAC species (Fig 3). The colony morphology on the HI01 sample plate remained uniform.

Each distinct colony morphology was subjected to acid-fast bacillus (AFB) and Gram staining. All pink morphologies were Gram negative and not AFB. The white morphologies from HI03 and HI04 were AFB and inconclusive by Gram staining (Fig 3). While NTM are considered Gram positive bacteria, mycolic acids have been reported to interfere with Gram staining methods, often leading to inconclusive results.

All distinct colony morphologies were also grown on 7H10-OADC containing malachite green (MG). The white morphologies from HI03 and HI04 along with the pink morphologies from HI01 and HI03 grew successfully. Only the pink morphology from HI04 failed to grow on 7H10-OADC-MG.

## Conclusion

In this study, isolated bacterial cultures from four environmental samples were sequenced with Illumina and MinION platforms. The Illumina reads produced assemblies fragmented into hundreds of contigs and were not sufficient on their own for our analytical goals. During the NTM workshop discussed here, community scientists including high school students and teachers successfully sequenced these same samples with the ONT MinION platform. The addition of long reads greatly improved the assembly results, allowing us to separate dual genomes, distinguish and identify plasmids, and map the location of species-identifier genes.

HI01 comprised a single circularized *Methylobacterium* chromosomal unit and up to five plasmids. HI02, though not complete, appeared to contain only a single chromosomal unit and had exclusively NTM DNA. By comparison, while HI03 and HI04 were each isolated as single colonies initially, each sample contained DNA of two distinct genomes with each having two *dnaA* annotations and genome sizes nearly twice the expected size of a single NTM genome. These data suggest that HI03 and HI04 contained both NTM and methylobacterial genomes. The finding of methylobacteria and NTM co-existence was surprising given prior reports suggesting that NTM and methylobacteria rarely occur together in the same environment [54] and never in the same biofilm [55]. A number of putative biofilm producing genes were found in the NTM genomes, including the non-ribosomal peptide synthetases *pstA1*, *pstB1*, *pstC2* [56, 57] and the methylobacterial genomes, including *sucA* [56], *pstA*, *pstB*, *pstC*, suggesting that all of the isolates may be capable of producing biofilms. Although we did not perform biofilm experiments as part of this study, some literature suggests that methylobacteria may interfere with NTM biofilms either by preventing adhesion to a surface [55] or degrading an existing NTM biofilm [58] but the exact compound or method remains unknown. If this is the case, it is possible that the methylobacteria in this study lacked the mechanism for preventing or degrading NTM biofilms resulting in the co-existing populations.

We explored the possibility of contamination at several steps of the protocol. Because the samples contained distinct *Methylobacterium* species, it is unlikely that these bacteria were introduced as a contaminant during the culturing step. It is also unlikely that methylobacteria was introduced during the workshop activity, because upon classification of the ONT and Illumina sequencing reads using Kraken [59], all read sets except those from HI02 contained a mixture of both species (results not shown).

Even though initial Sanger sequencing of the *rpoB* gene revealed that all four samples were pure NTM, the whole genome sequencing and assembly results indicate that methylobacteria were present at the time of DNA extraction; however, it is unclear how the methylobacteria survived the NTM-specific culturing steps. The Sanger NTM *rpoB* call of sample HI01 may indicate that NTM was present in the initial isolation, but at low abundance, and may have been outcompeted by methylobacteria during subsequent culture preparations for DNA extraction. Since the Sanger NTM *rpoB* sequencing method involves targeted amplification with primers optimized for NTM, it is possible to get amplification of the NTM *rpoB* gene even in the presence of other non-NTM species.

We used NTM culturing processes that select for mycobacteria using two antimicrobial agents: malachite green and cetylpyridinium chloride (CPC). Malachite green is a potent antibacterial agent included in Middlebrook media because mycobacteria are highly resistant to its killing effects, while other species are not. In previous studies, *Mycobacterium tuberculosis* was found to be capable of surviving on malachite green due to the gene encoding liproprotein signal peptidase II (*lspA*) [60]. All of the isolates in this study, including the methylobacteria, had at least one copy of this gene. Though it is unclear if *lspA* is typical of methylobacterial genomes or if it performs the same functional role outside of *M*. *tuberculosis*, the presence of this gene could partially explain the survival of methylobacteria in Middlebrook media. CPC is an antiseptic compound that kills non-acid-fast bacteria [61] e.g., methylobacteria. Though no specific gene was identified, the methylobacteria grown in this study appear to be resistance to CPC as well. Because of the assumed antagonistic relationship between NTM and methylobacteria, to the best of our knowledge, survival of methylobacteria in mycobacterial culturing conditions has not been tested.

After recovering the DNA sequences from both mycobacteria and methylobacteria, the samples were recultured and the pink (methylobacteria) and white/clear (mycobacteria) were separated successfully in culture. It is possible that the NTM facilitated methylobacterial survival during the selection processes through a symbiotic relationship. It is also possible that methylobacteria in Hawai’i are uniquely able to co-exist with NTM.

To better understand the genomic content of NTM and methylobacteria in this study, the assemblies were divided by species and assessed individually for annotations that could contribute to their ability to survive within showerheads, kitchen sinks, and garden hoses. Each of the environmental samples contained genes for acquiring ammonia (*amt*), a rich nitrogen source essential for cellular survival [62]. NTM assemblies tended to have *amt* whereas the methylobacteria all contained *amtB* except for HI01-Me that had both genes (Table 2). HI02-NTM and HI03-Me had complete sets of *chdABC* which is required for the C-8 oxidation mechanism to degrade caffeine. The methylobacterium in HI04-Me had two of the three genes and may not be capable of forming the entire dehydrogenase enzyme [63]. The presence of genes *nicA* and *nicB* suggest that these methylobacterial strains may be capable of degrading nicotinic acid [64]. Additional laboratory experimentation is needed to determine whether these microbes can use ammonia, caffeine, or nicotinic acid as carbon or nitrogen sources.

Each genome contained the same gene sets for maintaining homeostasis of magnesium (*corA*, *mgtA*) [65, 66] and divalent cations (*mntH*) [67]. Our analysis suggests that NTM and methylobacteria rely on different mechanisms to acquire iron. The NTM had *irtA*, which encodes a membrane protein that facilitate the import of iron-bound siderophores in iron-deprived environments such as those inside activated macrophages [68]. In contrast, the methylobacterial genomes contained *iscA* whose protein product binds iron directly and may work in conjunction with the ferredoxin family (*fdx* genes) also found among the annotations [22, 69].

All genomes included the arsenic *arsBC* genes encoding a membrane pump that transports arsenic out of the cell [70]. Arsenic is found ubiquitously in natural environments and the *arsBC* genotype is common in bacteria isolated from arsenic-contaminated sewage and creeks [71]. The gene *acrC* was also present in all but HI03-NTM and may function as an additional pump for arsenic export [28].

Among the annotations were numerous genes associated with resistance to antibiotics such as β-lactams, isoniazid, and ethambutol. While the β-lactamases encoded by *ampC* and *bla* directly inactivate β-lactam antibiotics [42, 43], some of the other identified genes encode efflux pumps that simply purge various drugs from the cell [49, 51]. All the samples used in this study were isolated from environmental sources where they are unlikely to have encountered these specific antibiotics. It is unclear whether these genes are remnants from previous exposure to antibiotics or if they serve different functions when in the environment. Regardless, the presence of these genes suggest that these environmental opportunistic pathogens are already equipped to survive antibiotic treatment when they infect a host; however, additional experimental analysis is required to test antibiotic tolerance.

To date, thousands of environmental samples have been collected across the Hawaiian Islands. In addition to these four samples that were sequenced by both long read and short read sequencing, we also plan to undertake short read genomic sequencing of hundreds of more NTM isolates, and to perform long read sequencing on a select number of these to produce high quality complete genome assemblies as described here. As more of these samples are fully sequenced and assembled, we aim to further elucidate the genomic diversity of Hawai’i environmental and clinical NTM, in order to better understand the high NTM infection burden in the state of Hawai’i and to shed further light on the co-existence of NTM and methylobacteria.

Our results show that NTM sequencing can be conducted successfully in a high school laboratory setting, and used as an efficient method to teach the concepts of microbial genomics. Through this workshop we have identified a suite of genes that may help environmental NTM survive a variety of environmental conditions, and the presence of antibiotic resistance genes suggests that NTM are already equipped to persist treatment in the event of causing an infection. We also found evidence of NTM co-existing with methylobacteria. It is unclear at this time whether the co-existence and co-isolation of mycobacteria and methylobacteria is unique among NTM in Hawai’i or whether it may contribute to the high incidence of NTM lung infections in the state. This work was made possible by the community scientists who collected thousands of samples from their local environments across Hawai’i and who, though this workshop, also contributed to the genomic sequencing. The sequencing activity was designed such that it can be reimplemented in high school classrooms or workshops in order to engage students and community members in the concepts of microbial genomics and opportunistic environmental pathogens, leading to novel insights for further research.

## Supporting information

S1 File Course materials. Material to reimplement workshop.
(PDF)

S2 File Expanded methods and results. Additional Methods and Results.
(DOCX)

## References

[1] van Ingen J, Obradovic M, Hassan M, Lesher B, Hart E, Chatterjee A, et al. Nontuberculous mycobacterial lung disease caused by Mycobacterium avium complex—disease burden, unmet needs, and advances in treatment developments. Expert Rev Respir Med. 2021;15(11):1387–401. doi: 10.1080/17476348.2021.1987891 34612115

[2] Hasan NA, Davidson RM, Epperson LE, Kammlade SM, Beagle S, Levin AR, et al. Population Genomics and Inference of Mycobacterium avium Complex Clusters in Cystic Fibrosis Care Centers, United States. Emerg Infect Dis. 2021;27(11):2836–46. doi: 10.3201/eid2711.210124 34670648 PMC8544995

[3] Davidson RM, Hasan NA, Epperson LE, Benoit JB, Kammlade SM, Levin AR, et al. Population Genomics of Mycobacterium abscessus from U.S. Cystic Fibrosis Care Centers. Ann Am Thorac Soc. 2021;18(12):1960–9. doi: 10.1513/AnnalsATS.202009-1214OC 33856965 PMC8641822

[4] Falkinham JO, 3rd. Nontuberculous mycobacteria from household plumbing of patients with nontuberculous mycobacteria disease. Emerg Infect Dis. 2011;17(3):419–24. 21392432 10.3201/eid1703.101510 PMC3166028

[5] Adjemian J, Olivier KN, Seitz AE, Holland SM, Prevots DR. Prevalence of nontuberculous mycobacterial lung disease in U.S. Medicare beneficiaries. Am J Respir Crit Care Med. 2012;185(8):881–6. doi: 10.1164/rccm.201111-2016OC 22312016 PMC3360574

[6] Honda JR, Hasan NA, Davidson RM, Williams MD, Epperson LE, Reynolds PR, et al. Correction: Environmental Nontuberculous Mycobacteria in the Hawaiian Islands. PLoS Negl Trop Dis. 2016;10(12):e0005200. doi: 10.1371/journal.pntd.0005200 27911953 PMC5135035

[7] Nelson ST, Robinson S, Rey K, Brown L, Jones N, Dawrs SN, et al. Exposure Pathways of Nontuberculous Mycobacteria Through Soil, Streams, and Groundwater, Hawai’i, USA. Geohealth. 2021;5(4):e2020GH000350. doi: 10.1029/2020GH000350 33855248 PMC8025848

[8] Parsons AW, Dawrs SN, Nelson ST, Norton GJ, Virdi R, Hasan NA, et al. Soil Properties and Moisture Synergistically Influence Nontuberculous Mycobacterial Prevalence in Natural Environments of Hawai’i. Appl Environ Microbiol. 2022;88(9):e0001822. doi: 10.1128/aem.00018-22 35435715 PMC9088257

[9] Honda JR, Chan YL, Tong EI, Kawatachi M, Virdi R, Norton GJ, et al. Inaugural nontuberculous mycobacterial lung disease education and research conference, Honolulu, Hawai’i, February 1–2, 2020. Microbes Infect. 2021;23(1):104763. doi: 10.1016/j.micinf.2020.09.009 33035706 PMC7536519

[10] ‘Aina-Informatics. https://www.nawaiekolu.org/ainainformatics. Accessed: 2022-01-01.”

[11] Oxford. Oxford Nanopore Technologies [Available from: https://nanoporetech.com/.

[12] Hendrix J, Epperson LE, Durbin D, Honda JR, Strong M. Intraspecies plasmid and genomic variation of Mycobacterium kubicae revealed by the complete genome sequences of two clinical isolates. Microb Genom. 2021;7(1). doi: 10.1099/mgen.0.000497 33355531 PMC8115904

[13] Epperson LE, Strong M. A scalable, efficient, and safe method to prepare high quality DNA from mycobacteria and other challenging cells. J Clin Tuberc Other Mycobact Dis. 2020;19:100150. doi: 10.1016/j.jctube.2020.100150 32154387 PMC7052505

[14] Jiang H, Lei R, Ding SW, Zhu S. Skewer: a fast and accurate adapter trimmer for next-generation sequencing paired-end reads. BMC Bioinformatics. 2014;15:182. doi: 10.1186/1471-2105-15-182 24925680 PMC4074385

[15] Wick RR, Judd LM, Holt KE. Performance of neural network basecalling tools for Oxford Nanopore sequencing. Genome Biol. 2019;20(1):129. doi: 10.1186/s13059-019-1727-y 31234903 PMC6591954

[16] Wick RR, Judd LM, Gorrie CL, Holt KE. Unicycler: Resolving bacterial genome assemblies from short and long sequencing reads. PLoS Comput Biol. 2017;13(6):e1005595. doi: 10.1371/journal.pcbi.1005595 28594827 PMC5481147

[17] Seemann T. Prokka: rapid prokaryotic genome annotation. Bioinformatics. 2014;30(14):2068–9. doi: 10.1093/bioinformatics/btu153 24642063

[18] Khademi S O’Connell J 3rd, Remis J, Robles-Colmenares Y, Miercke LJ, Stroud RM. Mechanism of ammonia transport by Amt/MEP/Rh: structure of AmtB at 1.35 A. Science. 2004;305(5690):1587–94. doi: 10.1126/science.1101952 15361618

[19] Yu CL, Kale Y, Gopishetty S, Louie TM, Subramanian M. A novel caffeine dehydrogenase in Pseudomonas sp. strain CBB1 oxidizes caffeine to trimethyluric acid. J Bacteriol. 2008;190(2):772–6. doi: 10.1128/JB.01390-07 17981969 PMC2223706

[20] Jimenez JI, Canales A, Jimenez-Barbero J, Ginalski K, Rychlewski L, Garcia JL, et al. Deciphering the genetic determinants for aerobic nicotinic acid degradation: the nic cluster from Pseudomonas putida KT2440. Proc Natl Acad Sci U S A. 2008;105(32):11329–34. doi: 10.1073/pnas.0802273105 18678916 PMC2516282

[21] Rodriguez GM, Smith I. Identification of an ABC transporter required for iron acquisition and virulence in Mycobacterium tuberculosis. J Bacteriol. 2006;188(2):424–30. doi: 10.1128/JB.188.2.424-430.2006 16385031 PMC1347291

[22] Ollagnier-de-Choudens S, Mattioli T, Takahashi Y, Fontecave M. Iron-sulfur cluster assembly: characterization of IscA and evidence for a specific and functional complex with ferredoxin. J Biol Chem. 2001;276(25):22604–7. doi: 10.1074/jbc.M102902200 11319236

[23] Hmiel SP, Snavely MD, Florer JB, Maguire ME, Miller CG. Magnesium transport in Salmonella typhimurium: genetic characterization and cloning of three magnesium transport loci. J Bacteriol. 1989;171(9):4742–51. doi: 10.1128/jb.171.9.4742-4751.1989 2548998 PMC210275

[24] Payandeh J, Li C, Ramjeesingh M, Poduch E, Bear CE, Pai EF. Probing structure-function relationships and gating mechanisms in the CorA Mg2+ transport system. J Biol Chem. 2008;283(17):11721–33. doi: 10.1074/jbc.M707889200 18276588

[25] Mishra AK, Batt S, Krumbach K, Eggeling L, Besra GS. Characterization of the Corynebacterium glutamicum deltapimB’ deltamgtA double deletion mutant and the role of Mycobacterium tuberculosis orthologues Rv2188c and Rv0557 in glycolipid biosynthesis. J Bacteriol. 2009;191(13):4465–72. doi: 10.1128/JB.01729-08 19395496 PMC2698491

[26] Kuroda M, Ohta T, Uchiyama I, Baba T, Yuzawa H, Kobayashi I, et al. Whole genome sequencing of meticillin-resistant Staphylococcus aureus. Lancet. 2001;357(9264):1225–40. doi: 10.1016/s0140-6736(00)04403-2 11418146

[27] Agranoff D, Monahan IM, Mangan JA, Butcher PD, Krishna S. Mycobacterium tuberculosis expresses a novel pH-dependent divalent cation transporter belonging to the Nramp family. J Exp Med. 1999;190(5):717–24. doi: 10.1084/jem.190.5.717 10477555 PMC2195619

[28] Fu HL, Meng Y, Ordonez E, Villadangos AF, Bhattacharjee H, Gil JA, et al. Properties of arsenite efflux permeases (Acr3) from Alkaliphilus metalliredigens and Corynebacterium glutamicum. J Biol Chem. 2009;284(30):19887–95. doi: 10.1074/jbc.M109.011882 19494117 PMC2740414

[29] Diorio C, Cai J, Marmor J, Shinder R, DuBow MS. An Escherichia coli chromosomal ars operon homolog is functional in arsenic detoxification and is conserved in gram-negative bacteria. J Bacteriol. 1995;177(8):2050–6. doi: 10.1128/jb.177.8.2050-2056.1995 7721697 PMC176848

[30] Rosen BP, Weigel U, Monticello RA, Edwards BP. Molecular analysis of an anion pump: purification of the ArsC protein. Arch Biochem Biophys. 1991;284(2):381–5. doi: 10.1016/0003-9861(91)90312-7 1703401

[31] Tsai KJ, Yoon KP, Lynn AR. ATP-dependent cadmium transport by the cadA cadmium resistance determinant in everted membrane vesicles of Bacillus subtilis. J Bacteriol. 1992;174(1):116–21. doi: 10.1128/jb.174.1.116-121.1992 1530844 PMC205684

[32] Gaballa A, Cao M, Helmann JD. Two MerR homologues that affect copper induction of the Bacillus subtilis copZA operon. Microbiology (Reading). 2003;149(Pt 12):3413–21. doi: 10.1099/mic.0.26225-0 14663075

[33] Sitthisak S, Knutsson L, Webb JW, Jayaswal RK. Molecular characterization of the copper transport system in Staphylococcus aureus. Microbiology (Reading). 2007;153(Pt 12):4274–83. doi: 10.1099/mic.0.2007/009860-0 18048940

[34] Andersson M, Mattle D, Sitsel O, Klymchuk T, Nielsen AM, Moller LB, et al. Copper-transporting P-type ATPases use a unique ion-release pathway. Nat Struct Mol Biol. 2014;21(1):43–8. doi: 10.1038/nsmb.2721 24317491 PMC3904665

[35] Wolschendorf F, Ackart D, Shrestha TB, Hascall-Dove L, Nolan S, Lamichhane G, et al. Copper resistance is essential for virulence of Mycobacterium tuberculosis. Proc Natl Acad Sci U S A. 2011;108(4):1621–6. doi: 10.1073/pnas.1009261108 21205886 PMC3029754

[36] Lee JY, Yang JG, Zhitnitsky D, Lewinson O, Rees DC. Structural basis for heavy metal detoxification by an Atm1-type ABC exporter. Science. 2014;343(6175):1133–6. doi: 10.1126/science.1246489 24604198 PMC4151877

[37] Fujisawa M, Kusumoto A, Wada Y, Tsuchiya T, Ito M. NhaK, a novel monovalent cation/H+ antiporter of Bacillus subtilis. Arch Microbiol. 2005;183(6):411–20. doi: 10.1007/s00203-005-0011-6 16021482

[38] Wang Y, Moore M, Levinson HS, Silver S, Walsh C, Mahler I. Nucleotide sequence of a chromosomal mercury resistance determinant from a Bacillus sp. with broad-spectrum mercury resistance. J Bacteriol. 1989;171(1):83–92. doi: 10.1128/jb.171.1.83-92.1989 2536669 PMC209558

[39] Shewchuk LM, Verdine GL, Nash H, Walsh CT. Mutagenesis of the cysteines in the metalloregulatory protein MerR indicates that a metal-bridged dimer activates transcription. Biochemistry. 1989;28(15):6140–5. doi: 10.1021/bi00441a002 2551364

[40] Volker U, Andersen KK, Antelmann H, Devine KM, Hecker M. One of two osmC homologs in Bacillus subtilis is part of the sigmaB-dependent general stress regulon. J Bacteriol. 1998;180(16):4212–8. doi: 10.1128/JB.180.16.4212-4218.1998 9696771 PMC107419

[41] Rensing C, Mitra B, Rosen BP. The zntA gene of Escherichia coli encodes a Zn(II)-translocating P-type ATPase. Proc Natl Acad Sci U S A. 1997;94(26):14326–31. doi: 10.1073/pnas.94.26.14326 9405611 PMC24962

[42] Lodge JM, Minchin SD, Piddock LJ, Busby SJ. Cloning, sequencing and analysis of the structural gene and regulatory region of the Pseudomonas aeruginosa chromosomal ampC beta-lactamase. Biochem J. 1990;272(3):627–31. doi: 10.1042/bj2720627 2125210 PMC1149754

[43] Barthelemy M, Peduzzi J, Bernard H, Tancrede C, Labia R. Close amino acid sequence relationship between the new plasmid-mediated extended-spectrum beta-lactamase MEN-1 and chromosomally encoded enzymes of Klebsiella oxytoca. Biochim Biophys Acta. 1992;1122(1):15–22. doi: 10.1016/0167-4838(92)90121-s 1633193

[44] Bentley J, Hyatt LS, Ainley K, Parish JH, Herbert RB, White GR. Cloning and sequence analysis of an Escherichia coli gene conferring bicyclomycin resistance. Gene. 1993;127(1):117–20. doi: 10.1016/0378-1119(93)90625-d 8486276

[45] Expression Kaur P. and characterization of DrrA and DrrB proteins of Streptomyces peucetius in Escherichia coli: DrrA is an ATP binding protein. J Bacteriol. 1997;179(3):569–75. 9006006 10.1128/jb.179.3.569-575.1997 PMC178733

[46] Guilfoile PG, Hutchinson CR. A bacterial analog of the mdr gene of mammalian tumor cells is present in Streptomyces peucetius, the producer of daunorubicin and doxorubicin. Proc Natl Acad Sci U S A. 1991;88(19):8553–7. doi: 10.1073/pnas.88.19.8553 1924314 PMC52547

[47] Choudhuri BS, Bhakta S, Barik R, Basu J, Kundu M, Chakrabarti P. Overexpression and functional characterization of an ABC (ATP-binding cassette) transporter encoded by the genes drrA and drrB of Mycobacterium tuberculosis. Biochem J. 2002;367(Pt 1):279–85. doi: 10.1042/BJ20020615 12057006 PMC1222852

[48] Colangeli R, Helb D, Sridharan S, Sun J, Varma-Basil M, Hazbon MH, et al. The Mycobacterium tuberculosis iniA gene is essential for activity of an efflux pump that confers drug tolerance to both isoniazid and ethambutol. Mol Microbiol. 2005;55(6):1829–40. doi: 10.1111/j.1365-2958.2005.04510.x 15752203

[49] Fujisaki S, Ohnuma S, Horiuchi T, Takahashi I, Tsukui S, Nishimura Y, et al. Cloning of a gene from Escherichia coli that confers resistance to fosmidomycin as a consequence of amplification. Gene. 1996;175(1–2):83–7. doi: 10.1016/0378-1119(96)00128-x 8917080

[50] De Rossi E, Branzoni M, Cantoni R, Milano A, Riccardi G, Ciferri O. mmr, a Mycobacterium tuberculosis gene conferring resistance to small cationic dyes and inhibitors. J Bacteriol. 1998;180(22):6068–71. doi: 10.1128/JB.180.22.6068-6071.1998 9811672 PMC107688

[51] Ramon-Garcia S, Martin C, De Rossi E, Ainsa JA. Contribution of the Rv2333c efflux pump (the Stp protein) from Mycobacterium tuberculosis to intrinsic antibiotic resistance in Mycobacterium bovis BCG. J Antimicrob Chemother. 2007;59(3):544–7. doi: 10.1093/jac/dkl510 17242035

[52] Son MS, Del Castilho C, Duncalf KA, Carney D, Weiner JH, Turner RJ. Mutagenesis of SugE, a small multidrug resistance protein. Biochem Biophys Res Commun. 2003;312(4):914–21. doi: 10.1016/j.bbrc.2003.11.018 14651958

[53] Chung YJ, Saier MH, Jr. Overexpression of the Escherichia coli sugE gene confers resistance to a narrow range of quaternary ammonium compounds. J Bacteriol. 2002;184(9):2543–5. 11948170 10.1128/JB.184.9.2543-2545.2002 PMC135002

[54] Falkinham JO 3rd, Williams MD, Kwait R, Lande L. Methylobacterium spp. as an indicator for the presence or absence of Mycobacterium spp. Int J Mycobacteriol. 2016;5(2):240–3. doi: 10.1016/j.ijmyco.2016.03.001 27242240

[55] Munoz Egea MC, Ji P, Pruden A, Falkinham Iii JO. Inhibition of Adherence of Mycobacterium avium to Plumbing Surface Biofilms of Methylobacterium spp. Pathogens. 2017;6(3). doi: 10.3390/pathogens6030042 28906463 PMC5617999

[56] Yamazaki Y, Danelishvili L, Wu M, Macnab M, Bermudez LE. Mycobacterium avium genes associated with the ability to form a biofilm. Appl Environ Microbiol. 2006;72(1):819–25. doi: 10.1128/AEM.72.1.819-825.2006 16391123 PMC1352297

[57] Wu CW, Schmoller SK, Bannantine JP, Eckstein TM, Inamine JM, Livesey M, et al. A novel cell wall lipopeptide is important for biofilm formation and pathogenicity of Mycobacterium avium subspecies paratuberculosis. Microb Pathog. 2009;46(4):222–30. doi: 10.1016/j.micpath.2009.01.010 19490829 PMC2691860

[58] Garcia-Coca M, Rodriguez-Sevilla G, Perez-Domingo A, Aguilera-Correa JJ, Esteban J, Munoz-Egea MC. Inhibition of Mycobacterium abscessus, M. chelonae, and M. fortuitum biofilms by Methylobacterium sp. J Antibiot (Tokyo). 2020;73(1):40–7. doi: 10.1038/s41429-019-0232-6 31481764

[59] Wood DE, Salzberg SL. Kraken: ultrafast metagenomic sequence classification using exact alignments. Genome Biol. 2014;15(3):R46. doi: 10.1186/gb-2014-15-3-r46 24580807 PMC4053813

[60] Banaei N, Kincaid EZ, Lin SY, Desmond E, Jacobs WR, Jr., Ernst JD. Lipoprotein processing is essential for resistance of Mycobacterium tuberculosis to malachite green. Antimicrob Agents Chemother. 2009;53(9):3799–802. 19596883 10.1128/AAC.00647-09 PMC2737861

[61] du Moulin GS, Kurt. Use of Cetylpyridinium Chloride in the Decontamination of Water for Culture of Mycobacteria. Applied and Environmental Microbiology. 1978;36:771–3. 727788 10.1128/aem.36.5.771-773.1978 PMC243135

[62] Zheng L, Kostrewa D, Berneche S, Winkler FK, Li XD. The mechanism of ammonia transport based on the crystal structure of AmtB of Escherichia coli. Proc Natl Acad Sci U S A. 2004;101(49):17090–5. doi: 10.1073/pnas.0406475101 15563598 PMC535379

[63] Vega FE, Emche S, Shao J, Simpkins A, Summers RM, Mock MB, et al. Cultivation and Genome Sequencing of Bacteria Isolated From the Coffee Berry Borer (Hypothenemus hampei), With Emphasis on the Role of Caffeine Degradation. Front Microbiol. 2021;12:644768. doi: 10.3389/fmicb.2021.644768 33889142 PMC8055839

[64] Tang H, Wang L, Meng X, Ma L, Wang S, He X, et al. Novel nicotine oxidoreductase-encoding gene involved in nicotine degradation by Pseudomonas putida strain S16. Appl Environ Microbiol. 2009;75(3):772–8. doi: 10.1128/AEM.02300-08 19060159 PMC2632140

[65] Maguire ME. MgtA and MgtB: prokaryotic P-type ATPases that mediate Mg2+ influx. J Bioenerg Biomembr. 1992;24(3):319–28. doi: 10.1007/BF00768852 1328179

[66] Park Y, Ahn YM, Jonnala S, Oh S, Fisher JM, Goodwin MB, et al. Inhibition of CorA-Dependent Magnesium Homeostasis Is Cidal in Mycobacterium tuberculosis. Antimicrob Agents Chemother. 2019;63(10). doi: 10.1128/AAC.01006-19 31383669 PMC6761525

[67] Domenech P, Pym AS, Cellier M, Barry CE 3rd, Cole ST. Inactivation of the Mycobacterium tuberculosis Nramp orthologue (mntH) does not affect virulence in a mouse model of tuberculosis. FEMS Microbiol Lett. 2002;207(1):81–6. doi: 10.1111/j.1574-6968.2002.tb11032.x 11886755

[68] Ryndak MB, Wang S, Smith I, Rodriguez GM. The Mycobacterium tuberculosis high-affinity iron importer, IrtA, contains an FAD-binding domain. J Bacteriol. 2010;192(3):861–9. doi: 10.1128/JB.00223-09 19948799 PMC2812465

[69] Balasubramanian R, Shen G, Bryant DA, Golbeck JH. Regulatory roles for IscA and SufA in iron homeostasis and redox stress responses in the cyanobacterium Synechococcus sp. strain PCC 7002. J Bacteriol. 2006;188(9):3182–91. doi: 10.1128/JB.188.9.3182-3191.2006 16621810 PMC1447454

[70] Meng YL, Liu Z, Rosen BP. As(III) and Sb(III) uptake by GlpF and efflux by ArsB in Escherichia coli. J Biol Chem. 2004;279(18):18334–41. doi: 10.1074/jbc.M400037200 14970228

[71] Saltikov CW, Olson BH. Homology of Escherichia coli R773 arsA, arsB, and arsC genes in arsenic-resistant bacteria isolated from raw sewage and arsenic-enriched creek waters. Appl Environ Microbiol. 2002;68(1):280–8. doi: 10.1128/AEM.68.1.280-288.2002 11772637 PMC126541

