# Supplementary material for: Complete genome assembly of Hawai’i environmental nontuberculous mycobacteria reveals unexpected co-isolation with methylobacteria
Source: PLoS One. 2023 Sep 13;18(9):e0291072. doi: 10.1371/journal.pone.0291072 (PMC10499228; doi:10.1371/journal.pone.0291072)
Supplement: S1 File — Material to reimplement workshop. (PDF) [file pone.0291072.s001.pdf]

Appendix 1: Course materials

Instructor Materials

Learning Objectives

- Reflect on the potential implications of environmental NTM being present in the local environment.
- Demonstrate safe and effective laboratory techniques.
- Describe MinION sequences and how it can be used to answer research questions.
- Be able to identify sequence mutations and predict the functional implications on the protein.

Safety Issues

Student protocols were designed to avoid contact with any harmful reagents or biological components.

Master Supply List

A list of supplies and catalogue numbers is provided below. The curriculum was designed to use only equipment found in a typical microbial teaching laboratory and materials available from Oxford Nanopore Technologies (ONT). In addition, minimal computational resources are required.

Bacterial Isolates

- 400µg High Molecular Weight DNA (per sample)
  - See Sample Collection and DNA Isolation Protocol below

DNA library preparation

- Pipettes: P10 recommended; P2 (optional)
- Thermocycler
  - Alternative: two heat blocks, one set to 30°C and the other set to 80°C
- Ice bucket with ice for incubation
- Microfuge

- Timer
- Rapid Barcoding Kit
    - ONT - SQK-RBK004
- Nuclease-free water (7.5µl per sample)
- Tubes (1 per sample + 1 per library)
    - 1.5ml Eppendorf DNA tubes or 0.2ml PCR tubes depending on thermocycler/heat block size

Sequencing

- MinION device
    - ONT - MIN-101B (MinION Mk1B)
- Flow Cell
    - ONT - FLO-MIN106 or ADP-FLG001
- Flow Cell Priming Kit
    - ONT - EXP-FLP002
- Flow Cell Wash Kit
    - ONT - EXP-WSH003
- Vortex
- Pipettes: P10, P20, P200, and P1000
- Nuclease-free water (4.5µl per library)
- Tube (1 per library)
    - 1.5ml Eppendorf DNA tubes or 0.2ml PCR tubes depending on thermocycler/heat block size
- Computer
    - USB3 port or adapter
    - 16GB RAM
    - 1 TB internal storage
- MinKNOW software to control MinION device
    - See current system requirements as these may change
- Alternative: ONT - MinION Mk1C
    - The MinION device, Computer, and MinKNOW software can be substituted for the MinION Mk1C. This is an all-in-one device that controls the sequencing run and displays results.
- iPads for streaming (optional)

Post-Sequencing Analysis

- Basecaller
    - There are several options for basecalling and each has different system requirements. The MinKNOW software (run from a computer or the MinION Mk1C) offers a fast-basecalling option for immediate results. External Guppy

software offers high-accuracy basecalling for improved results. Guppy can be run on CPUs but this can be very slow so a NVIDIA GPU-based system is recommended. Compute resources may also be accessed through Amazon Web Services (AWS), Google Cloud Platform (GCP), EPI2ME, or other cloud-based platforms.

- Assembler
  - Many assemblers are available. For long-read only assembly, we recommend Canu. If Illumina data is also available, we recommend hybrid assembly with Unicycler.

Bioinformatics Tutorial

- Computers for students
- Internet access

Methods

Bioinformatics Tutorial

There are many different types of analysis that can be performed after DNA sequencing. The purpose of this tutorial is to introduce students to gene identification, sequence alignment, and protein visualization.

The tutorial titled “Nontuberculous mycobacteria: A Genetic Analysis using Online Bioinformatics Tools” can be accessed at http://www.stronglab.org/ntmlesson/.

Handouts

Instructor and student handouts can be found below. We recommend that the Bioinformatics Tutorial be distributed to students digitally to allow students to copy and paste the provided sequences.

‘Āina-Informatics

MinION Sequencing Lab Protocol Overview
Rapid Barcoding Kit[1]

Protocol step	What’s actually happening
Testing the pore activity of the flow cell using the MinKNOW software.	The MinION detects which **nanopores** on the **flow cell** are available by sequencing DNA test fragments that are present in the storage buffer. The minimum number of available pores recommended is 800.
Barcoding the DNA in each library separately	Each piece of genomic DNA within a **library** is enzymatically cleaved and a pre-assigned **barcode** is attached to each end of every resulting fragment. 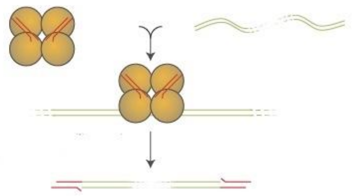
Pooling the samples	Each barcoded library is then combined in equal proportions to be processed and sequenced together on a single flow cell.
Attaching the sequencing adapters	An enzyme attaches **sequencing adapters** to each end of every barcoded DNA fragment. A single **motor protein** comes pre-attached to each sequencing adapter. 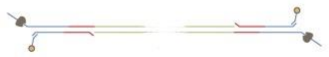
Priming the flow cell	The flow cell must be primed to receive the final DNA sequencing library. This step removes the storage buffer and provides ATP for the motor proteins.
Preparing the final DNA sequencing library	The barcoded, pooled and adapted DNA is then mixed with loading beads, which help to distribute the DNA fragments evenly across the sensor array during loading.
Loading the DNA library	The final library is loaded directly onto the sensor array for sequencing.

[1] Adapted for use with Oxford Nanopore Technology’s Rapid Barcoding Kit (SQK-RBK004)

Key Terms

Nanopore: A synthetic transmembrane channel through which one strand of each DNA fragment to be sequenced passes. Characteristic changes in the electrical field induced by each passing base are detected and recorded by the MinION sequencer.

Flow cell: A piece of nanotechnology which contains an array of nanopores upon which DNA samples to be sequenced are loaded. It transmits the electrical signals from the nanopores to the MinION sequencer.

Library: A sequencing library consists of all the DNA fragments originating from a single sample that will be sequenced simultaneously. A “pooled” or “multiplexed” library consists of all the DNA fragments originating from multiple source libraries that are combined in order to be sequenced simultaneously.

Barcode: A sequence of DNA manufactured to be unique enough that, when attached to a subset of DNA fragments within a pooled library, can be used to algorithmically sort the resulting sequences into their original source libraries.

Multiplexing: Combining multiple libraries to be sequenced simultaneously on a single flow cell.

Sequencing adapter: A sequence of synthetic DNA which attaches to each barcoded DNA fragment and allows the fragment to interface with the nanopore.

Motor protein: A protein attached to each sequencing adapter, and therefore to each DNA fragment to be sequenced, which ratchets one strand of the DNA fragment into the nanopore one base at a time.

[1] Adapted for use with Oxford Nanopore Technology’s Rapid Barcoding Kit (SQK-RBK004)

Teacher's Prep Guide

Required equipment and reagents:

- Micropipettes and tips
- MinION device and flow cell*
- Computer with MinKNOW software
- Thermocycler
- Lo-Bind Eppendorf tubes
- PCR tubes
- Molecular biology grade water
- Ethernet or wifi

* Included in the MinION Basic Starter Pack

Recommended equipment:

- Vortex
- Microcentrifuge
- DNA quantification system (i.e. Qubit)

Required kits:

- Oxford Nanopore Rapid Barcoding Kit (SQK-RBK004)
- Oxford Nanopore Flow Cell Priming Kit (EXP-FLP002)*

Pre-lab preparation:

- Source high molecular weight genomic DNA from one or more microbial cultures. If extracting DNA from generic microbial cultures, we recommend using a commercial kit optimized for soil or stool (i.e. Machery-Nagel).
- Quantify the DNA to obtain its concentration. The minimum concentration needed for this workflow is around 50 ng/μL per library.
- For detailed equipment and reagent handling procedures, refer to the Oxford Nanopore Community (free customer account required) for manufacturer protocols. The ʻĀina-Informatics protocols provided here are abridged for student use.

Set your stations:

- The number of student stations will depend on the number of libraries you intend to prepare. These protocols are optimized for 3 or fewer libraries.
- For each station:

P-10 or P-20 micropipette and tips	1
Molecular biology grade water	>10.0 μL
Genomic DNA sample	>7.5 μL
PCR tube	1
Fragmentation Mix RB01-12 (one per station)	1 tube

You are now ready to begin library preparation for a multiplexed MinION whole genome sequencing run.

[1] Adapted for use with Oxford Nanopore Technology's Rapid Barcoding Kit (SQK-RBK004)

‘Āina-Informatics

MinION Sequencing Library Preparation Protocol
Rapid Barcoding Kit[1]

<table>
<tr><th>Step</th><th>Protocol Action</th><th>What’s actually happening</th></tr>
<tr><td colspan="3">Testing pore activity of the flow cell</td></tr>
<tr><td>1</td><td>Connect the MinION to the computer and install the flow cell.

Using the MinKNOW software, run a pore test experiment.</td><td>The MinION detects which pores are actively sequencing using DNA test fragments in the storage buffer.</td></tr>
<tr><td colspan="3">Fragment the genomic DNA and attach the barcodes</td></tr>
<tr><td>2</td><td>In a small PCR tube, prepare the following recipe for each library:

<table><tr><th>Reagent</th><th>Volume</th></tr><tr><td>400 ng genomic DNA</td><td>7.5 µL</td></tr><tr><td>Fragmentation Mix RB01-12 (one for each sample)</td><td>2.5 µL</td></tr><tr><td>Total</td><td>10.0 µL</td></tr></table></td><td>Each DNA barcode is attached to a transposase, an enzyme which can cleave your genomic DNA into fragments, and then attach the barcode sequence to the ends of each resulting fragment.</td></tr>
<tr><td>3</td><td>Using a thermocycler, incubate the reaction at the following temperatures:

<table><tr><th>Temperature</th><th>Duration</th></tr><tr><td>30 °C</td><td>1:00 min</td></tr><tr><td>80 °C</td><td>1:00 min</td></tr><tr><td>4 °C</td><td>HOLD</td></tr></table></td><td>The transposome is activated by raising the reaction temperature to 30 °C. The enzyme is then deactivated at 80 °C, and the product is preserved by holding the temperature at 4 °C.</td></tr>
</table>

[1] Adapted for use with Oxford Nanopore Technology’s Rapid Barcoding Kit (SQK-RBK004)

Pool the barcoded samples		
4	In a 1.5 mL Eppendorf tube, combine equal volumes of each library for a total volume of **10 μL**.	Running multiple libraries together on a single flow cell, or **multiplexing**, makes the most efficient use of the flow cell.
**Attach the sequencing adapters**		
5	To your DNA, add **1 μL** of **Rapid Adapter (RAP)**.<br><br>Mix gently by flicking the tube, and spin down.<br><br>Incubate the reaction at **room temperature** for **5 min**.	Each **sequencing adapter** comes pre-attached with a single **motor protein**. An enzyme attaches an adapter to each end of every barcoded fragment of DNA in your reaction.
**Continue on to priming and loading the flow cell**		

Key Terms

Library: A sequencing library consists of all the DNA fragments originating from a single sample that will be sequenced simultaneously. A "pooled" or "multiplexed" library consists of all the DNA fragments originating from multiple source libraries that are combined in order to be sequenced simultaneously.

Barcode: A sequence of DNA manufactured to be unique enough that, when attached to a subset of DNA fragments within a pooled library, can be used to algorithmically sort the resulting sequences into their original source libraries.

Multiplexing: Combining multiple libraries to be sequenced simultaneously on a single flow cell.

Sequencing adapter: A sequence of synthetic DNA which attaches to each barcoded DNA fragment and allows the fragment to interface with the nanopore.

Motor protein: A protein attached to each sequencing adapter, and therefore to each DNA fragment to be sequenced, which ratchets one strand of the DNA fragment into the nanopore one base at a time.

‘Āina-Informatics

Loading the MinION Flow Cell Protocol[1]

<table>
<tr><th>Step</th><th>Protocol Action</th><th>What’s actually happening</th></tr>
<tr><td colspan="3">Prime the MinION flow cell</td></tr>
<tr><td>1</td><td>Prepare the Priming Mix by adding 30 µL of Flush Tether (FLT) directly into 1 tube of Flush Buffer (FB). Mix by vortexing.</td><td>This Priming Mix contains ATP which powers the motor protein.</td></tr>
<tr><td>2</td><td>Open the priming port.

Set a P-1000 pipette to 200 µL and insert the tip into the priming port. Using the pipette wheel, draw back a small amount of storage buffer into the pipette tip.</td><td>This step is to remove any air, which is damaging to the nanopores in the sensor array, from the priming port.</td></tr>
<tr><td>3</td><td>Slowly load 800 µL of the Priming Mix in through the priming port, avoiding the introduction of air bubbles. Wait 5 min.</td><td>The Priming Mix forces the yellow storage buffer off the sensor array and into the waste channel.</td></tr>
<tr><td colspan="3">Prepare the library for loading onto the flow cell</td></tr>
<tr><td>4</td><td>In a 1.5 mL Eppendorf tube, prepare the library for loading according to the following recipe:

<table>
<tr><th>Reagent</th><th>Volume</th></tr>
<tr><td>Sequencing Buffer (SQB)</td><td>34.0 µL</td></tr>
<tr><td>Loading Beads (LB), mixed before use</td><td>25.5 µL</td></tr>
<tr><td>Nuclease-free water</td><td>4.5 µL</td></tr>
<tr><td>Prepared DNA library</td><td>11.0 µL</td></tr>
<tr><td>Total</td><td>75.0 µL</td></tr>
</table>
</td><td>The Loading Beads settle quickly and must be resuspended immediately before use. The beads help to distribute the DNA fragments evenly across the sensor array during the loading process.</td></tr>
</table>

[1] Adapted for use with Oxford Nanopore Technology’s Flow Cell Priming Kit (EXP-FLP002), which is included with the Rapid Sequencing Kit (SQK-RAD004) and the Rapid Barcoding Kit (SQK-RBK004).

**Complete the final step in priming the flow cell**		
**5**	Gently lift the **SpotON sample port cover**.<br><br>Slowly load **200 µL** of the **Priming Mix** into the priming port, pausing for buffer coming up from the **SpotON port** to recede before loading more.	This step ensures that when the library is loaded, it is continuous with the Priming Mix, and therefore no air is introduced into the flow cell.
**Load the DNA library**		
**6**	Mix the prepared library by gently pipetting up and down to resuspend the loading beads.<br><br>Add **75 µL** of your prepared library in a drop-wise fashion into the SpotON sample port, ensuring that each drop flows into the port before adding the next.<br><br>Return the SpotON sample port and priming port covers to their closed positions.	Your sample is now in contact with the nanopores in the sensor array and can be sequenced.
**Start the sequencing run**		
**7**	In the MinKNOW software, begin the sequencing run by providing a run ID, sample ID, library prep kit information and a location for data storage.<br><br>Select fast basecalling and barcoding options if applicable.	The software will begin detecting the electrical signals generated by bases passing through each active pore and send the data to the cloud for basecalling.

Parts of a MinION Flow Cell

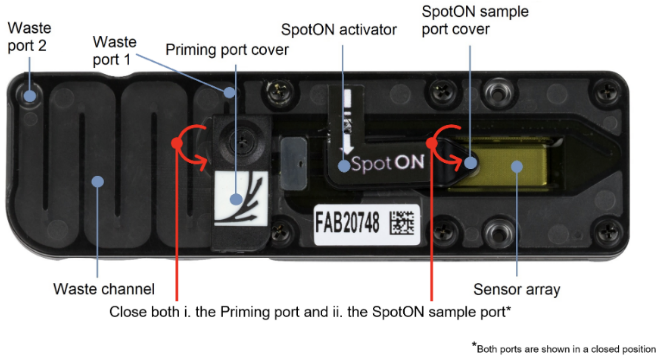

ʻĀina-Informatics

Washing the MinION Flow Cell Protocol[1]

Step	Protocol Action	What's actually happening
**Prepare the flow cell wash mix**		
**1**	If sequencing is still ongoing, note the number of active pores in MinKNOW and stop data acquisition.<br><br>In a 1.5 mL Eppendorf tube, prepare the **Flow Cell Wash Mix** according to the following recipe, mix by pipetting and store on ice until needed.<br><br>**Reagent** / **Volume**<br>Wash Mix (WMX): 2.0 μL<br>Wash Diluent (DIL): 398.0 μL<br>**Total**: **400.0 μL**	The **Flow Cell Wash Mix** contains DNase I, an enzyme that digests double-stranded DNA, diluted in a buffer which maximizes the activity of DNase I.<br><br>This solution will remove any remaining DNA on the **sensor array** from the previous sequencing run.
**Remove waste from the flow cell waste channel**		
**2**	With both the **priming port** and **SpotOn port** closed, use a P-1000 pipette to remove all the fluid in the **waste channel** from **waste port 1**.	This step should only remove fluid from the waste channel, not from the sensor array.
**Flush the previous libraries from the flow cell sensor array**		
**3**	Open the priming port. Set a P-1000 pipette to 200 μL and insert the tip into the priming port. Using the pipette wheel, draw back a small amount of fluid into the pipette tip.	This step is to remove any air, which is damaging to the nanopores in the sensor array, from the priming port.
**4**	Slowly load **400 μL** of the **Flow Cell Wash Mix** in through the priming port, avoiding the introduction of air bubbles. Close the priming port and wait **60 min**.	The Flow Cell Wash Mix digests up to 99.9% of the DNA from the previous run.

[1] Adapted for use with Oxford Nanopore Technology's Flow Cell Wash Kit (EXP-WSH004).

**Remove waste from the flow cell waste channel**		
**5**	With both the priming port and SpotOn port closed, use a P-1000 pipette to remove all the fluid in the waste channel from waste port 1.	This step again removes fluid from the waste channel, not from the sensor array.
**To reuse the flow cell right away**		
**6**	**To prepare the flow cell for short term storage, proceed to step 7.**<br><br>Use the **Priming and Loading the MinION Flow Cell Protocol** to re-prime the flow cell for immediate reuse.	The washed flow cell can be reused immediately.
**To prepare the flow cell for short term storage**		
**7**	Open the priming port. Set a P-1000 pipette to 200 µL and insert the tip into the priming port. Using the pipette wheel, draw back a small amount of fluid into the pipette tip.	This step is to remove any air, which is damaging to the nanopores in the sensor array, from the priming port.
**8**	Add **500 µL** room temperature **Storage Buffer (S)** in through the priming port, avoiding the introduction of bubbles. Close the priming port.	The Storage Buffer preserves the sensor array and allows for subsequent pore checks.
**Remove remaining waste from flow cell and store**		
**9**	Use a P-1000 pipette to remove all the fluid in the waste channel from waste port 1.	Once all waste is removed, store the flow cell at **4 °C**.

Parts of a MinION Flow Cell

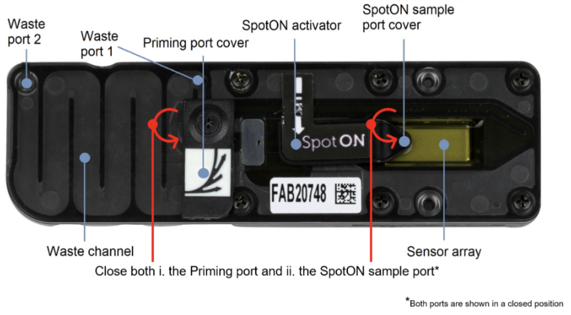

Nontuberculous mycobacteria: A Genetic Analysis Using Online Bioinformatics Tools

Overview

This lesson asks you to compare gene sequences between one wild-type and one of a variety of mutant nontuberculous mycobacterial strains. You will identify mutations as single-nucleotide polymorphisms (SNPs) and then make an inference on whether your variant strain will impact the activity of the protein or antibiotics that target the protein.

Objectives

By the end of this activity you will be able to:

1) Infer mycobacterial species identity, using sequence search tools including BLAST, https://blast.ncbi.nlm.nih.gov/Blast.cgi

2) Compare environmental NTM sequences to clinical sequences, using multiple sequence alignment tools, including CLUSTAL, https://www.ebi.ac.uk/Tools/msa/clustalo/

3) Identify and explain what a single-nucleotide polymorphism (SNP) is when comparing two gene sequences.

4) Navigate online scientific tools to translate DNA into polypeptide sequences, using tools such as GeneMarkS http://exon.gatech.edu/GeneMark/genemarks.cgi , and to compare and contrast wild-type and variant polypeptide sequences using CLUSTAL, https://www.ebi.ac.uk/Tools/msa/clustalo/.

5) Determine whether your given SNP will result in sense, missense, or nonsense mutations in the resulting amino acid sequence.

6) Hypothesize whether a SNP will impact antibiotic activity, using information derived from protein databases including the Protein Data Bank (PDB), https://www.rcsb.org

Part 1

A patient has just been diagnosed with a mycobacterial infection. In order to treat this effectively, you need to know the species. You decide single gene sequencing is adequate for this.

Please use BLAST, to search DNA databases to determine the species of mycobacteria, based on the DNA sequence.

```
>unknown species
atgcgcggcaacactggaggaccgatcttggcagtctctcgccagactaagaccgataac
gcaactactaactccgtacctggggcccctagccgactttccttcgccaagctgcgtgaa
ccgcttgcggttcccggcctgctcgatgtgcagacggagtcctttgaatggctggttgga
tcgccgcgctggcgtgaggttgcgactgcacgcggtgaggtgaacccgaccggcggcctt
gaggagatcctcacggagctttcgccgatcgaagacttctccggctcgatgtcgctgtcg
ttcagcgacccgcgcttcgacgaggtcaaggcgcccgtcgacgagtgcaaagacaaggac
atgacgtacgcggccccgctgttcgtcacggccgagttcatcaacaacaacaccggtgag
atcaagagccagacggtcttcatgggtgatttcccgatgatgaccgatatgggcaccttc
atcatcaacggcaccgagcgcgtggtcgtgtcgcagctcgtccgttcgccgggtgtctac
ttcgacgagagcatcgacaagtcgaccgagaagaccctgcatagcgtcaaggtcatcccc
ggccgcggtgcctggctcgagttcgacgtcgacaagcgcgacaccgtcggcgtccgcatc
gaccgcaagcgccgccagccggtcaccgtgctgctgaaggcgctgggctggaccaacgag
cagatcgtcgagcgcttcgggttctccgagatcatgatgggcaccctggagaaggacaac
atcgccggtcccgacgaggcgttgctggacatctaccgcaagctgcgcccgggcgagccg
ccgaccaaggagtcggcgcaggccctgctggagaacctgttcttcaaggagaagcgttac
gacctggcccgcgtgggtcggtacaaggtgaacaagaagctgggcctgggcggcaccaat
ccggctcaggtgaccaccaccaccctcaccgaggaagacgtcgtcgccaccatcgagtac
ctggtgcgcctgcacgagggccagaccacgatgaccgcccccggtggcgtcgaggtgccg
gtggatgtggacgacatcgaccacttcggtaaccgtcgcctgcgtaccgtcggcgagctg
attcagaaccagatccgggtcggcctgtcccgtatggagcgcgtcgtgcgtgagcgcatg
accacgcaggacgtcgaggcgatcaccccgcagaccctgatcaacatccgtcccgtcgtg
gcggcgatcaaggagttcttcggaaccagccagctgtcgcagttcatggaccagaacaac
ccgctgtcgggcctgacccacaagcgtcgt
```

Limit search to: Mycobacteria (taxid:85007) and Sequences from Type Material

Part 2

You asked the patients to sample their home, to see if any swabs came up NTM culture positive. Some locations came up positive, which you then sequenced using the Sanger method. BLAST each of the following sequences to determine potential species.

```
>Environmental_Location_1
1     acgacgcttg tgggtcagac ccgacagcgg gttgttctgg tccatgaact gcgacagctg
61    gctggttccg aagaactcct tgatcgccgc caccacggga cggatgttga tcagggtctg
121   cggggtgatc gcctcgacgt cctgcgtggt catgcgctcg cgcacgacac gttccatacg
```

```
181   ggacaggccg acccggatct ggttctggat cagctcgccg acggtacgca ggcgacggtt
241   accgaagtgg tcgatgtcgt ccacgtccac cggcacctcg acgccgccgg gggcggtcat
301   cgtggtctgg ccctcgtgca ggcgcaccag atactcgatg gtggcgacga catcttcctc
361   ggtgagggtg gtggtggtca cctgagccgg attggtgccg cccaggccca gcttcttgtt
421   caccttgtac cgacccacgc gggccaggtc gtaacgcttc tccttgaaga acaggttctc
481   cagcagggcc tgcgccgact ccttggtcgg cggctcgccc gggcgcagct tgcggtagat
541   gtccagcaac gcctcgtcgg gaccggcgat gttgtccttc tccagggtac ccatcatgat
601   ctcggagaac ccgaagcgct cgacgatctg ctcgttggtc cagcccagcg ccttcagcag
661   cacggtgacc ggctgacggc gcttgcggtc gatgcggacg ccgacggtgt cgcgcttgtc
721   gacgtcgaac tcgagccagg caccgcggcc ggggatgacc ttgacgctgt gcagggtctt
781   ctcggtcgac ttgtcgatgc tctcgtcgaa gtagacaccc ggcgaacgga cgagctgcga
841   gacgaccacg cgctcggtgc cgttgatgat gaaggtgccc atgtcggtca tcatcgggaa
901   atcacccatg aagaccgtct ggctcttgat ctcaccggtg ttgttgttga tgaactcggc
961   cgtgacgaac agcggggccg cgtacgtcat gtccttgtct ttgcactcgt cgacgggcgc
1021  cttgacctcg tcgaagcgcg ggtcgctgaa cgacagcgac atcgagccgg agaagtcttc
1081  gatcggcgaa agctccgtga ggatctcctc aaggccgccg gtcgggttca cctcaccgcg
1141  tgcagtcgca acctcacgcc agcgcggcga tccaaccagc cattcaaagg actccgtctg
1201  cacatcgagc aggccgggaa ccgcaagcgg ttcacgcagc ttggcgaagg aaagtcggct
1261  aggggcccca ggtacggagt tagtagttgc gttatcggtc ttagtctggc gagagactgc
1321  caagatcggt cctccagtgt tgccgcgcat
```

```
>Environmental_Location_2
1     acgacgcttg tgggtcagac ccgacagcgg gttgttctgg tccatgaact gcgacagctg
61    gctggttccg aagaactcct tgatcgccgc cacgacggga cggatgttga tcagggtctg
121   cggagtgatc gcctcgacgt cctgagtggt catgcgctcg cgcacgacgc gctccatacg
181   cgacaggccg acccggatct ggttctggat cagctcgccg acagtacgca gacgacggtt
241   accgaagtga tcgatgtcgt cgacctcgac ggggacctcg aggccgccgg gggcggtcat
301   cgtggtctgg ccctcgtgca gacgcaccag gtactcgatg gtggcgacga cgtcttcctc
361   ggtgagcgtg gtggcagtca ccagagccgg gttggcaccg ccaagaccca gcttcttgtt
421   caccttgtac cgacccacgc gggccaggtc gtaacgcttc tccttgaaga acaggttctc
481   cagcagggcc tgcgcggact ccttggtcgg cggctcgccc ggacgcagct tgcggtagat
541   gtccagcagc gcctcgtcgg gaccggcgat gttgtccttc tccagggtcc ccatcatgat
601   ctcggagaac ccgaaacgct cgacgatctg ctcgttggtc cagccgagtg ccttcagcag
661   cacggtgacg ggctgacgac gcttgcgatc gatgcgcaca cccacggtgt cgcgcttgtc
721   gacatcgaac tcgagccatg caccacggcc ggggatgacc ttgacgctgt gcagggtctt
781   ctcggtcgac ttatcgatgt tctcgtcgaa gtagacaccc ggcgaacgga cgagctgcga
841   cacgaccacg cgctcggtgc cgttgatgat gaaggtgccc atctcggtca tcatcgggaa
901   atcacccatg aagaccgtct ggctcttgat ctcgccggtg ttgttgttga tgaactcggc
961   cgtgacgaac agcggagccg cgtacgtcat gtccttgtct ttgcactcgt cgacgggcgc
1021  cttgacctcg tcgaagcgcg ggtcgctgaa agacagcgac atcgagcccg agaagtcctc
1081  gatcggcgaa agctccgtga ggatctcctc aaggccgccg gtcgggttca cctcaccgcg
1141  agcagttgca acttcacgcc accggggtga gccaaccagc cattcaaagg aatccgtctg
1201  cacatccagc aggccgggaa ccgcgagcgg ttcacgcagc ttggcgaagg aaagtcggct
1261  aggggcccca ggtacggagt tagtagttgc gttatcggtc ttagtctggc gagagactgc
1321  caagatcggt cctccagtgt tgccgcgcat
```

```
>Environmental_Location_3
1     atcttggcag tctctagcca gagcacagcg aacgctaaca ccaataactc cgtcccagga
61    gcaccaaacc gagtttcctt tgccaagctc cgcgaaccgc ttgaggttcc ggggctgctc
121   gacgttcaga cggattcttt cgactggctc gtgggttcgg atgaatggcg gcagaaggcc
181   gtcgatcgcg gtgagaccga ccccaagggc ggcctcgaag aggtgctcga agagctctcg
241   ccgatcgagg atttctcggg ctcgatgtcg ctgagcttct ccgacccgcg cttcgacgag
```

```
301   gtcaaggcgc cggtcgacga gtgcaaagac aaggacatga cgtacgcggc cccgctgttc
361   gtcacggccg agttcatcaa caacaacacc ggtgagatca agagccagac ggtcttcatg
421   ggtgacttcc cgatgatgac cgagaagggc accttcatca tcaacggcac cgagcgtgtc
481   gtggtgagcc agctcgtgcg ctctcccggt gtgtacttcg acgagagcat cgacaagtcc
541   accgagaaga cgctgcacag cgtcaaggtg atccccggcc gcggtgcgtg gctcgagttc
601   gacgtcgaca agcgcgacac cgtcggtgtg cgtatcgacc gcaagcgccg ccagccggtc
661   accgtgctgc tcaaggcgct cggttggacc aacgagcaga tcaccgagcg cttcggcttc
721   tccgagatca tgatgggcac cctggagaag gacagcaccg ccggtcccga cgaggcgctg
781   ctggacatct accgcaagct gcgtccgggc gagccgccga ccaaggagtc cgcgcagacc
841   ctgctggaga acctgttctt caaggagaag cgctacgacc tggcccgcgt cggccgctac
901   aaggtcaaca agaagctggg cctgaacgcc ggccagccga tcacgtcgtc gaccctcacc
961   gaggaagacg tcgtcgccac catcgagtac ctggtgcgcc tgcacgaggg ccagaccacg
1021  atgaccgtcc ccggcggcgt cgaggtcccg gtcgaggtgg acgacatcga ccacttcggt
1081  aaccgtcgtc tgcgtaccgt gggtgagctg atccagaacc agatccgggt cggcctgtcc
1141  cgcatggagc gcgtcgtgcg tgagcgcatg accacccagg acgtcgaggc gatcacgccg
1201  cagaccctga tcaacatccg tcccgtcgtg gcggcgatca aggagttctt cggcaccagc
1261  cagctgtcgc agttcatgga ccagaacaac ccgctgtcgg gtctgaccca caagcgtcg

>Environmental_Location_4
1     atcttggcag tctctagcca gagcaagtca gcgaacgcta tcaccaataa ctccgtccca
61    ggagcaccga accgagtttc atttgccaag ctccgtgaac cgcttgaggt tccggggcta
121   ctcgacgttc agaccgattc cttcgactgg ctcgtcggtg cggatgaatg gcggcagaag
181   gccgtcgatc gcggcgagac cgaccccaag ggcggcctcg aagaggtgct cgaagagctc
241   tccccgatcg aggatttctc gggctcgatg tcgctgagct tctccgaccc gcgcttcgac
301   gaggtcaaag ctccggtcga cgagtgcaaa gacaaggaca tgacgtacgc agccccgctg
361   ttcgtcacgg ccgagttcat caacaacaac accggtgaga tcaagagcca gacggtcttc
421   atgggtgact tcccgatgat gaccgagaag ggcaccttca tcatcaacgg caccgagcgt
481   gtcgtggtga gccagctcgt gcgctctccc ggtgtgtact tcgacgagag catcgacaag
541   tccaccgaga agacgctgca cagcgtcaag gtgatccccg gccgcggtgc gtggctggag
601   ttcgacgtcg acaagcgcga caccgtcggt gtgcgtatcg accgcaagcg tcgtcagccg
661   gtcaccgtgc tgctgaaggc gctgggctgg accaacgagc agatcaccga gcgcttcggc
721   ttctccgaga tcatgatggg caccctggag aaggacagca ccgccggtcc cgacgaggcg
781   ctgctggaca tctaccgcaa gctgcgtccg ggcgagccgc cgaccaagga gtccgcgcag
841   accctgctgg agaacctgtt cttcaaggag aagcgctacg acctggcccg cgtgggccgc
901   tacaaggtca acaagaagct gggcctgaac gccggccagc cgatcacgtc gtcgactctg
961   accgaggaag acgtcgtcgc caccatcgag tacctggtgc gcctgcacga gggccagacc
1021  acgatgaccg tccccggcgg cgtcgaggtc ccggtcgagg tggacgacat cgaccacttc
1081  ggtaaccgtc gtctgcgcac cgtgggcgag ctgatccaga accagatccg cgtcggcctg
1141  tcccgcatgg agcgcgtcgt gcgtgagcgc atgaccaccc aggacgtcga ggcgatcacc
1201  ccgcagaccc tgatcaacat ccgtcccgtc gtggcggcga tcaaggagtt cttcggaacg
1261  tcgcagctgt cgcagttcat ggatcagaac aacccgctgt cgggtctgac ccacaagcgt
1321  cgt

>Environmental_Location_5
1     acgacgcttg tgggtcaggc ccgacagcgg gttgttctgg tccatgaact gcgacagctg
61    gctggttccg aagaactcct tgatcgccgc cacgacggga cggatgttga tcagggtctg
121   cggggtgatc gcctcgacgt cctgcgtggt catgcgctca cgcacgacgc gctccatacg
181   ggacaggccg acccggatct ggttctgaat cagctcgccg acggtacgca ggcgacggtt
241   accgaagtgg tcgatgtcgt ccacatccac cggcacctcg acgccaccgg gggcggtcat
301   cgtggtctgg ccctcgtgca ggcgcaccag gtactcgatg gtggcgacga cgtcttcctc
361   ggtgagggtg gtggtggtca cctgagccgg attggtgccg cccaggccca gcttcttgtt
421   caccttgtac cgacccacgc gggccaggtc gtaacgcttc tccttgaaga acaggttctc
```

```
481   cagcagggcc tgcgccgact ccttggtcgg cggctcgccc gggcgcagct tgcggtagat
541   gtccagcaac gcctcgtcgg gaccggcgat gttgtccttc tccagggtgc ccatcatgat
601   ctcggagaac ccgaagcgct cgacgatctg ctcgttggtc cagcccagcg ccttcagcag
661   cacggtgacc ggctggcggc gcttgcggtc gatgcggacg ccgacggtgt cgcgcttgtc
721   gacgtcgaac tcgagccagg caccgcggcc ggggatgacc ttgacgctat gcagggtctt
781   ctcggtcgac ttgtcgatgc tctcgtcgaa gtagacaccc ggcgaacgga cgagctgcga
841   cacgaccacg cgctcggtgc cgttgatgat gaaggtgccc atatcggtca tcatcgggaa
901   atcacccatg aagaccgtct ggctcttgat ctcaccggtg ttgttgttga tgaactcggc
961   cgtgacgaac agcggggccg cgtacgtcat gtccttgtct ttgcactcgt cgacgggcgc
1021  cttgacctcg tcgaagcgcg ggtcgctgaa cgacagcgac atcgagccgg agaagtcttc
1081  gatcggcgaa agctccgtga ggatctcctc aaggccgccg gtcgggttca cctcaccgcg
1141  tgcagtcgca acctcacgcc agcgcggcga tccaaccagc cattcaaagg actccgtctg
1201  cacatcgagc aggccgggaa ccgcaagcgg ttcacgcagc ttggcgaagg aaagtcggct
1261  aggggcccca ggtacggagt tagtagttgc gttatcggtc ttagtctggc gagagactgc
1321  caagatcggt cctccagtgt tgccgcgcat
```

Based on this information, which location warrants further investigation, by whole genome sequencing?

Part 3
You are interested in variations in protein sequences of these strains as well, corresponding to the RNA polymerase beta subunit. Translate the environmental isolate sequences to amino acid sequences, using GeneMarkS.

Part 4
Perform a Multiple Sequence Alignment on the translated protein sequences, to examine amino acid variation of the rpoB protein, in the environmental NTM, using CLUSTAL.

Part 5
In addition to being an essential protein involve in RNA transcription, the RNA polymerase beta protein is an important drug target, in both NTM and M. tuberculosis.

Search the Protein Data Bank (PDB), using the Environmental Isolate 5 sequence, to see if protein structures with similar sequences have been determined.

References

Epperson, L. Elaine, and Michael Strong. "A scalable, efficient, and safe method to prepare high quality DNA from mycobacteria and other challenging cells." *Journal of clinical tuberculosis and other mycobacterial diseases* 19 (2020): 100150.
