# Supplementary material for: Complete genome assembly of Hawai’i environmental nontuberculous mycobacteria reveals unexpected co-isolation with methylobacteria
Source: PLoS One. 2023 Sep 13;18(9):e0291072. doi: 10.1371/journal.pone.0291072 (PMC10499228; doi:10.1371/journal.pone.0291072)
Supplement: S2 File — Additional Methods and Results. (DOCX) [file pone.0291072.s002.docx]

Supplemental Methods

*Genome assembly*

Following the workshop, Illumina reads were assembled alone using Unicycler v0.4.4 (1) and ONT reads were assembled alone using Canu v2.2 (2). The ONT and Illumina reads were assembled together using Unicycler v0.4.4 (1) to construct the hybrid assemblies. We assessed assembly quality by the number of contigs and errors in the sequence such that fewer was considered better. To predict the number of errors, the Illumina reads were aligned to the respective Illumina-only or hybrid assembly using Bowtie2 and the number of positions where more than 20% of the reads supported the non-reference allele were counted.

*Student Evaluation of Program Activity*

After the activity, students were given an evaluation of the workshop using statements on a scale of 1-5 where 5 represented ‘strongly agree’ and 1 corresponded to ‘strongly disagree.’ The rating was calculated as the weighted average of the scores: the number of students who chose a score was multiplied by the numerical score itself then added to the sum for a statement. The statement sum was then divided by 100. Students also provided feedback through a free-style comment box.

Supplemental Results

*Assembly*

Assemblies constructed with only Illumina reads were fragmented into 219 to 585 contigs with 875 to 1,822 predicted errors. Hybrid assembly, i.e., combining both the Illumina and ONT reads, reduced the number of contigs to a range of 9 to 29 and the number of errors to range from 114 to 409 (Table 2). Only the hybrid assemblies were used for further analysis.

Table S1: Comparison of Illumina-only and Hybrid assemblies

|  | **Illumina-only Assembly** | | | **Hybrid Assembly** | | |
| --- | --- | --- | --- | --- | --- | --- |
| ***ID*** | **Length** | **# Contigs** | **# Errors** | **Length** | **# Contigs** | **# Errors** |
| *HI01* | 6,379,691 | 250 | 1,342 | 6,542,248 | 9 | 114 |
| *HI02* | 7,120,479 | 228 | 880 | 7,201,782 | 23 | 226 |
| *HI03* | 11,459,188 | 219 | 875 | 11,625,743 | 20 | 140 |
| *HI04* | 12,936,430 | 585 | 1,822 | 13,302,866 | 29 | 409 |

*Annotations*

Though not mentioned in the text, all three NTM genomes along with the *Me. populi* in HI01-Me and HI03-Me contained a gene encoding a cocaine esterase. This esterase breaks down the cocaine molecule and may help these organisms use the cocaine from the coca plant as an alternative source of carbon and nitrogen, though this was not tested here (3).

Table S2:

| Function | Gene | HI01-Me | HI03-Me | HI04-Me | HI02-NTM | HI03-NTM | HI04-NTM | Citation |
| --- | --- | --- | --- | --- | --- | --- | --- | --- |
| Metabolism |  |  |  |  |  |  |  |  |
| Cocaine breakdown | *cocE* | ✓ | ✓ |  | ✓ | ✓ | ✓ | (3) |

*Instructions to implement activity*

In addition to our new genomic findings, this study incorporated a significant educational component. The MinION is a cutting-edge, portable, lower-cost, and readily accessible academic tool because students can complete a protocol with little prior laboratory experience, witness the sequencer in action, and generate high-quality, real-world data. However, to date, reported uses of MinION sequencing in pre-college settings are rare (4).

During such activities, it is essential that groups remain small so that each student has ample opportunity to interact with the sample prep. Large groups with too many students working on a single sample prep reduce student participation in similar activities (5). Instructors wishing to implement MinION sequencing in the classroom or workshop are highly encouraged to utilize barcoding and pooling of samples so that all students can have hands-on experience while reducing the required number of flow cells, and the consequent cost. The Rapid Barcoding Kit (SQK-RBK004) includes 12 barcodes, allowing up to 12 student groups to share a single flow cell.

Our NTM MinION Workshop curriculum may be modeled in other schools or workshops either as a single event or divided into modules across multiple time blocks as needed. The Modules are:

Module 1 (1 hour): Introduce project, provide background information. Instructors may use the first hour to introduce the background information, including the origin of the samples, and explain how DNA was extracted. Discussions on environmental microbes and DNA sequencing can be included.

Module 2 (1 hour): Instructions for activity and laboratory safety. The next hour should be dedicated to introducing laboratory techniques and safe practices. Students will have the opportunity to familiarize themselves with the laboratory space, practice pipetting, and review the protocol with the instructor.

Module 3 (1-2 hours): Sequencing activity. Students will follow the protocol and record the data. Completion time will vary depending on skill level. It is also recommended to conduct a short debriefing session afterward to answer remaining questions and to discuss the downstream analysis process.

Example handouts are provided in Supplemental 1.

*Student Evaluation*

Students evaluated the workshop by rating their agreement with five statements. When asked whether the laboratory demonstration was useful and informative, 16 (72.7%) participants agreed strongly and 6 (27.3%) agreed for an overall rating of 4.73 out of 5. Most participants strongly agreed (14; 63.6%) or agreed (7; 31.8%) that the laboratory information was presented well but one student (4.5%) felt neutral about this statement. Most participants strongly agreed (14; 63.6%) or agreed (6; 27.3%) that the workshop discussions were helpful while two students (9.1%) felt neutral towards this statement. (Supplemental 2 Table 2).

Comments expressed a general positive attitude towards the activity. Some examples are given below:

“I absolutely loved this conference… It was so engaging and informative. The presentations – awesome, the workshops- amazing.”

“The workshop was so fun!”

“It was good to learn techniques/methods for conducting experiments!”

Table S3: Student evaluation of workshop activity. The number of students and their rating agreement per evaluation statement.

| *Statement* | *Strongly agree*  *(5)* | *Agree*  *(4)* | *Neutral*  *(3)* | *Disagree*  *(2)* | *Strongly disagree*  *(1)* | *Avg. Rating* |
| --- | --- | --- | --- | --- | --- | --- |
| *The laboratory demonstrations were useful and informative* | 16 (72.7%) | 6 (27.3%) | 0 | 0 | 0 | 4.73 |
| *The laboratory information was presented in an effective manner* | 14 (63.6%) | 7 (31.8%) | 1 (4.5%) | 0 | 0 | 4.59 |
| *The laboratory and workshop discussions were helpful* | 14 (63.6%) | 6 (27.3%) | 2 (9.1%) | 0 | 0 | 4.55 |
| *The computer tutorial was useful and informative* | 15 (68.2%) | 4 (18.2%) | 3 (13.6%) | 0 | 0 | 4.55 |
| *The computer tutorial was presented in an effective manner* | 17 (77.3%) | 3 (13.6%) | 2 (9.1%) | 0 | 0 | 4.68 |

1. Wick RR, Judd LM, Gorrie CL, Holt KE. Unicycler: Resolving bacterial genome assemblies from short and long sequencing reads. PLoS Comput Biol. 2017;13(6):e1005595.

2. Koren S, Walenz BP, Berlin K, Miller JR, Bergman NH, Phillippy AM. Canu: scalable and accurate long-read assembly via adaptive k-mer weighting and repeat separation. Genome Res. 2017;27(5):722-36.

3. Bresler MM, Rosser SJ, Basran A, Bruce NC. Gene cloning and nucleotide sequencing and properties of a cocaine esterase from Rhodococcus sp. strain MB1. Appl Environ Microbiol. 2000;66(3):904-8.

4. Falkinham JO, 3rd. Nontuberculous mycobacteria from household plumbing of patients with nontuberculous mycobacteria disease. Emerg Infect Dis. 2011;17(3):419-24.

5. ‘Aina-Informatics.
