# Supplementary material for: Complete genome assembly of Hawai’i environmental nontuberculous mycobacteria reveals unexpected co-isolation with methylobacteria
Source: PLoS One. 2023 Sep 13;18(9):e0291072. doi: 10.1371/journal.pone.0291072 (PMC10499228; doi:10.1371/journal.pone.0291072)
Supplement: Attachment — Submitted filename: Comments.docx [file pone.0291072.s003.docx]

Dear Editor,

The manuscript titled “Complete Genome Assembly of Hawai’i Environmental Nontuberculous Mycobacteria Reveals Unexpected Co-Isolation with Methylobacteria” is an interesting study using community scientists and MinION based sequencing in identifying the co-inhabitation of NTM and methylobacteria in Hawai. The scientific work is highly commendable and could be considered for publication. It is noteworthy, that the authors used community scientist partnership , bench top sequencer to identify a epidemiological cause. The authors have validated the MinION using Illumina and microbiological phenotype characterization authenticating their findings.

As, point- of care diagnostics are most needed in health care, usage of bench top sequencers could pave way for epidemiological , diagnostics use in resource limited settings with appropriate trainings and validations.

I have the following minor queries.

Queries

1. Hawai being a tourist hotspot, are there any reports or whether the community scientist s in this study evaluated traveller loads from tuberculosis endemic countries especially in the 4 sample areas collected in this study
2. Where there any tuberculosis cases reported from the households or their contacts in the places of sample collection The authors have validated the MinION using Illumina and microbiological phenotype characterization authenticating their findings.
3. Was co-inhabitation of methylobacteria caused any TB disease in those areas during the sample collection period or post collection if any follow-up was done.
