# Supplementary material for: Complete genome assembly of Hawai’i environmental nontuberculous mycobacteria reveals unexpected co-isolation with methylobacteria
Source: PLoS One. 2023 Sep 13;18(9):e0291072. doi: 10.1371/journal.pone.0291072 (PMC10499228; doi:10.1371/journal.pone.0291072)
Supplement: Attachment — Submitted filename: Comments for PLoSOne.doc [file pone.0291072.s004.doc]

The manuscript entitled “Complete Genome Assembly of Hawai’i Environmental Nontuberculous Mycobacteria Reveals Unexpected Co-Isolation with Methylobacteria” is an interesting and well written manuscript, where the authors collected thousands of environmental samples for sequencing from a Hawai’i one of the highest incidence of NTM lung disease. They also found evidence of co-occurring Methylobacteria, revealed from the sequencing data, suggesting that in some cases Methylobacteria and NTM may coexist in the same niche.
