# Supplementary material for: Complete genome assembly of Hawai’i environmental nontuberculous mycobacteria reveals unexpected co-isolation with methylobacteria
Source: PLoS One. 2023 Sep 13;18(9):e0291072. doi: 10.1371/journal.pone.0291072 (PMC10499228; doi:10.1371/journal.pone.0291072)
Supplement: Attachment — Submitted filename: Response to Reviewers.doc [file pone.0291072.s005.doc]

6/13/2023

Re: Revision of “Complete Genome Assembly of Hawai’i Environmental Nontuberculous Mycobacteria Reveals Unexpected Co-Isolation with Methylobacteria.”

Dear *PLOS ONE* editors,

We are grateful to the Reviewers for their insightful comments and helpful suggestions to our manuscript. We have addressed each of the Reviewers’ comments below and revised the manuscript accordingly. We are excited to potentially publish this article in *PLOS ONE* and thank you for your consideration.

**REVIEWER #1**:

**Comment 1:** The authors mention that thousands of isolates were collected and four were sequenced. It is not clear why the authors selected only four isolates and why these particular isolates were selected. Further detail should be given on this aspect, as well as on whether there are plans for further sequencing of other isolates collected.

**Response:**

The text at lines 107-112 in the main text has been modified to include our reasoning for choosing these four particular samples: “Bacterial samples were collected from the Hawaiʻi community science project following methods previously described [6]. Four of these samples were selected for whole genome sequencing based on their unexpected pink pigmentation, which is typical of methylobacteria but not of NTM. Initial species identifications were determined through single locus PCR amplification and Sanger sequencing of the *rpoB* gene, which resulted in a mycobacterial identification for each sample. The samples were named HI01-04.”

Our future intention is to continue sequencing of the samples collected by the community science project and this has been added to the main text on lines 398-405: “To date, thousands of environmental samples have been collected across the Hawaiian Islands. In addition to these four samples that were sequenced by both long read and short read sequencing, we also plan to undertake short read genomic sequencing of hundreds of more NTM isolates, and to perform long read sequencing on a select number of these to produce high quality complete genome assemblies as described here. As more of these samples are fully sequenced and assembled, we aim to further elucidate the genomic diversity of Hawai’i environmental and clinical NTM, in order to better understand the high NTM infection burden in the state of Hawai’i and to shed further light on the co-existence of NTM and methylobacteria.”

**REVIEWER #4**:

**Comment 1:** Earlier studies have shown that biofilms play a very important role in preventing the co-existence of NTM and methylobacteria (PMID: 28906463, PMID: 31481764). Did the authors investigate the biofilm forming genes in the current study?

**Response:**

In the main document, text was added on lines 319-328 to discuss the presence of biofilm producing genes: “The finding of methylobacteria and NTM co-existence was surprising given prior reports suggesting that NTM and methylobacteria rarely occur together in the same environment [55] and never in the same biofilm [54]. A number of putative biofilm producing genes were found in the NTM genomes, including the non-ribosomal peptide synthetases *pstA1, pstB1, pstC2* [56,57] and the methylobacterial genomes, including *sucA* [56]*, pstA, pstB, pstC,* suggesting that all of the isolates may be capable of producing biofilms. Some literature suggests that methylobacteria interfere with NTM biofilms either by preventing adhesion to a surface [55] or degrading an existing NTM biofilm [58] but the exact compound or method remains unknown. If this is the case, it is possible that the methylobacteria in this study lacked the mechanism for preventing or degrading NTM biofilms resulting in the co-existing populations.”

**Comment 2:** Line no. 180, modify as ‘Gram stained as per manufacturer’s instructions’

**Response:** The text on line 186 was changed from “Gram stained (Fluka Analytical) per manufacturer’s instruction” to “Gram stained (Fluka Analytical) as per manufacturer’s instruction” with the addition of the word “as” in response to the reviewer’s comment.

**Comment 3:** Reference number 10 can be given in a proper format

**Response:** Additional information including website url and access data have been added to reference number 10 in the References list. Line 461 originally read “10. ‘Aina-Informatics.” and has been updated to “10. ‘Aina-Informatics. https://www.nawaiekolu.org/ainainformatics. Accessed: 2022-01-01.”

**REVIEWERS #2 OR #3**:

**Comment 1:** Hawaii being a tourist hotspot, are there any reports or whether the community scientists in this study evaluated traveller loads from tuberculosis endemic countries especially in the 4 sample areas collected in this study. Where there any tuberculosis cases reported from the households or their contacts in the places of sample collection. The authors have validated the MinION using Illumina and microbiological phenotype characterization authenticating their findings. Was co-inhabitation of methylobacteria caused any TB disease in those areas during the sample collection period or post collection if any follow-up was done.

**Response:** We thank the reviewer for these comments all related to tuberculosis. Respectively, we stress that this study was strictly a broad NTM environmental survey to characterize the genomic diversity of environmental NTM; thus, clinical data was not requested or collected as there was no patient participation here, tuberculosis or NTM or otherwise.

Community scientists in this project did not evaluate traveller loads from tuberculosis endemic countries, as this was not a study aim. Since the bacteria that cause tuberculosis (*M. tuberculosis* – an obligate human pathogen species that cannot sustain long periods in the environment) are distinct from NTM (environmental bacteria that can reside efficiently in the environment) we believe that that the environmental NTM is not seeded by travelers, but instead that NTM infections are predominantly acquired from NTM that are already present in the environmental locations.

We are not aware of instances where methylobacteria contributes directly to tuberculosis or NTM disease. Mycobacterial diseases are typically diagnosed by microbiologic and molecular methods that identify the presence of mycobacteria (*M. tuberculosis* or NTM), but not necessarily other infecting microorganisms.

Of note, we report a change of institution for co-senior author, Dr. Jennifer Honda and have noted this update on the Facepage and where applicable.

Also of note, we added references 55-58 to the manuscript to address reviewer comments.

Thank you again for your consideration for publication in *PLOS ONE*.

Sincerely,

Jo Hendrix

Jo Hendrix, Ph.D.

and

Michael Strong, Ph.D.

Associate Professor

Center for Genes, Environment and Health

National Jewish Health, Denver, CO, 80206

[StrongM@NJHealth.org](mailto:StrongM@NJHealth.org) , 303-270-2782
