# Supplementary material for: Complete genome assembly of Hawai’i environmental nontuberculous mycobacteria reveals unexpected co-isolation with methylobacteria
Source: PLoS One. 2023 Sep 13;18(9):e0291072. doi: 10.1371/journal.pone.0291072 (PMC10499228; doi:10.1371/journal.pone.0291072)
Supplement: Attachment — Submitted filename: Minor comments.docx [file pone.0291072.s006.docx]

Dear Editor,

The author’s have provided answers to the queries raised. However, the answer to the question raised by reviewer 1 about biofilm formation remains unclear from the author’s response.

1. Please report whether biofilm formation genes were identified in the current study
2. Please report whether any biofilm formations studies were done – it seems that authors did not perform this assay. Please rephrase , Some literature suggests that methylobacteria interfere with NTM biofilms either by preventing adhesion to a surface [55] or degrading an existing NTM biofilm [58] but the exact compound or method remains unknown. If this is the case, it is possible that the methylobacteria in this study lacked the mechanism for preventing or degrading NTM biofilms resulting in the co-existing populations.” Accordingly.
