# Supplementary material for: Complete genome assembly of Hawai’i environmental nontuberculous mycobacteria reveals unexpected co-isolation with methylobacteria
Source: PLoS One. 2023 Sep 13;18(9):e0291072. doi: 10.1371/journal.pone.0291072 (PMC10499228; doi:10.1371/journal.pone.0291072)
Supplement: Attachment — Submitted filename: Response to Reviewers.doc [file pone.0291072.s007.doc]

8/07/2023

Re: Revision of “Complete Genome Assembly of Hawai’i Environmental Nontuberculous Mycobacteria Reveals Unexpected Co-Isolation with Methylobacteria.”

Dear *PLOS ONE* editors,

We are grateful to the Reviewers for their insightful comments and helpful suggestions to our manuscript. We have addressed the Reviewer’s comments below and revised the manuscript accordingly. We are excited to potentially publish this article in *PLOS ONE* and thank you for your consideration.

**REVIEWER #2**:

**Comment 1:** Please report whether biofilm formation genes were identified in the current study.

**Response:**

We had added revised text regarding potential biofilm genes on lines 566-569 of the manuscript: “A number of putative biofilm producing genes were found in the NTM genomes, including the non-ribosomal peptide synthetases *pstA1, pstB1, pstC2* [56,57] and the methylobacterial genomes, including *sucA* [56]*, pstA, pstB, pstC,* suggesting that all of the isolates may be capable of producing biofilms.”

**Comment 2:** Please report whether any biofilm formations studies were done – it seems that authors did not perform this assay. Please rephrase , Some literature suggests that methylobacteria interfere with NTM biofilms either by preventing adhesion to a surface [55] or degrading an existing NTM biofilm [58] but the exact compound or method remains unknown. If this is the case, it is possible that the methylobacteria in this study lacked the mechanism for preventing or degrading NTM biofilms resulting in the co-existing populations.”

**Response:**

We appreciate the comment. Biofilm assays were beyond the study scope of this particular project, as this was a genomic focused study and educational experience for students who did not have the capacity or lab safety measures in place in the high school lab to address NTM biofilms. While Methylobacteria is BSL1, a biofilm assay to compare the two could not have been done by the students since NTM requires BSL2.

In response to the reviewer’s comment, we have revised the text on line 569-574 from “Some literature suggests that methylobacteria interfere with NTM biofilms either by preventing adhesion to a surface [55] or degrading an existing NTM biofilm [58] but the exact compound or method remains unknown. If this is the case, it is possible that the methylobacteria in this study lacked the mechanism for preventing or degrading NTM biofilms resulting in the co-existing populations.” to now read “Although we did not perform biofilm experiments as part of this study, some literature suggests that methylobacteria may interfere with NTM biofilms either by preventing adhesion to a surface [55] or degrading an existing NTM biofilm [58] but the exact compound or method remains unknown. If this is the case, it is possible that the methylobacteria in this study lacked the mechanism for preventing or degrading NTM biofilms resulting in the co-existing populations.”

Thank you again for your consideration for publication in *PLOS ONE*.

Sincerely,

Jo Hendrix

Jo Hendrix, Ph.D.

and

Michael Strong, Ph.D.

Associate Professor

Center for Genes, Environment and Health

National Jewish Health, Denver, CO, 80206

[StrongM@NJHealth.org](mailto:StrongM@NJHealth.org) , 303-270-2782
