# Supplementary material for: Complete genome assembly of Hawai’i environmental nontuberculous mycobacteria reveals unexpected co-isolation with methylobacteria
Source: PLoS One. 2023 Sep 13;18(9):e0291072. doi: 10.1371/journal.pone.0291072 (PMC10499228; doi:10.1371/journal.pone.0291072)
Supplement: Peer review history

# Peer review: Decision Letter 0

**DOI:** 10.1371/journal.pone.0291072.r001

**Authors:** Divakar Sharma

17 May 2023

PONE-D-23-05502Complete Genome Assembly of Hawai’i Environmental Nontuberculous Mycobacteria Reveals Unexpected Co-Isolation with MethylobacteriaPLOS ONE

Dear Dr. Hendrix,

Thank you for submitting your manuscript to PLOS ONE. After careful consideration, we feel that it has merit but does not fully meet PLOS ONE’s publication criteria as it currently stands. Therefore, we invite you to submit a revised version of the manuscript that addresses the points raised during the review process.

ACADEMIC EDITOR: Revision Requested

Please submit your revised manuscript by Jul 01 2023 11:59PM. If you will need more time than this to complete your revisions, please reply to this message or contact the journal office at plosone@plos.org. When you're ready to submit your revision, log on to https://www.editorialmanager.com/pone/ and select the 'Submissions Needing Revision' folder to locate your manuscript file.

Please include the following items when submitting your revised manuscript:

- A rebuttal letter that responds to each point raised by the academic editor and reviewer(s). You should upload this letter as a separate file labeled 'Response to Reviewers'.
- A marked-up copy of your manuscript that highlights changes made to the original version. You should upload this as a separate file labeled 'Revised Manuscript with Track Changes'.
- An unmarked version of your revised paper without tracked changes. You should upload this as a separate file labeled 'Manuscript'.

If you would like to make changes to your financial disclosure, please include your updated statement in your cover letter. Guidelines for resubmitting your figure files are available below the reviewer comments at the end of this letter.

If applicable, we recommend that you deposit your laboratory protocols in protocols.io to enhance the reproducibility of your results. Protocols.io assigns your protocol its own identifier (DOI) so that it can be cited independently in the future. For instructions see: https://journals.plos.org/plosone/s/submission-guidelines#loc-laboratory-protocols. Additionally, PLOS ONE offers an option for publishing peer-reviewed Lab Protocol articles, which describe protocols hosted on protocols.io. Read more information on sharing protocols at https://plos.org/protocols?utm_medium=editorial-email&utm_source=authorletters&utm_campaign=protocols.

We look forward to receiving your revised manuscript.

Kind regards,

Divakar Sharma

Academic Editor

PLOS ONE

Journal requirements:

When submitting your revision, we need you to address these additional requirements.

1. Please ensure that your manuscript meets PLOS ONE's style requirements, including those for file naming. The PLOS ONE style templates can be found at

https://journals.plos.org/plosone/s/file?id=wjVg/PLOSOne_formatting_sample_main_body.pdf and

https://journals.plos.org/plosone/s/file?id=ba62/PLOSOne_formatting_sample_title_authors_affiliations.pdf

2. Please review your reference list to ensure that it is complete and correct. If you have cited papers that have been retracted, please include the rationale for doing so in the manuscript text, or remove these references and replace them with relevant current references. Any changes to the reference list should be mentioned in the rebuttal letter that accompanies your revised manuscript. If you need to cite a retracted article, indicate the article’s retracted status in the References list and also include a citation and full reference for the retraction notice.

[Note: HTML markup is below. Please do not edit.]

Reviewers' comments:

Reviewer's Responses to Questions

**Comments to the Author**

1. Is the manuscript technically sound, and do the data support the conclusions?

The manuscript must describe a technically sound piece of scientific research with data that supports the conclusions. Experiments must have been conducted rigorously, with appropriate controls, replication, and sample sizes. The conclusions must be drawn appropriately based on the data presented. 

Reviewer #1: Yes

Reviewer #2: Yes

Reviewer #3: Yes

Reviewer #4: Yes

**********

2. Has the statistical analysis been performed appropriately and rigorously? 

Reviewer #1: N/A

Reviewer #2: I Don't Know

Reviewer #3: Yes

Reviewer #4: Yes

**********

3. Have the authors made all data underlying the findings in their manuscript fully available?

The PLOS Data policy requires authors to make all data underlying the findings described in their manuscript fully available without restriction, with rare exception (please refer to the Data Availability Statement in the manuscript PDF file). The data should be provided as part of the manuscript or its supporting information, or deposited to a public repository. For example, in addition to summary statistics, the data points behind means, medians and variance measures should be available. If there are restrictions on publicly sharing data—e.g. participant privacy or use of data from a third party—those must be specified.

Reviewer #1: Yes

Reviewer #2: Yes

Reviewer #3: Yes

Reviewer #4: Yes

**********

4. Is the manuscript presented in an intelligible fashion and written in standard English?

PLOS ONE does not copyedit accepted manuscripts, so the language in submitted articles must be clear, correct, and unambiguous. Any typographical or grammatical errors should be corrected at revision, so please note any specific errors here.

Reviewer #1: Yes

Reviewer #2: Yes

Reviewer #3: Yes

Reviewer #4: Yes

**********

5. Review Comments to the Author

Please use the space provided to explain your answers to the questions above. You may also include additional comments for the author, including concerns about dual publication, research ethics, or publication ethics. (Please upload your review as an attachment if it exceeds 20,000 characters)

Reviewer #1: The authors describe the details related to sequencing 4 NTM isolates from various sources (kitchen sink, showerhead, garden hose, and a beach showerhead) which were collected as part of a community science effort led by high school students. The findings represent a detailed analysis of the sequence data, along with the findings suggestive of concomitant methylobacteria. This finding is interesting and represents a potential avenue for further study, along with some of the other findings. The efforts at capacity building in the high school and community are admirable, particularly the efforts to teach high school students how to perform genetic sequencing.

Comments:

The authors mention that thousands of isolates were collected and four were sequenced. It is not clear why the authors selected only four isolates and why these particular isolates were selected. Further detail should be given on this aspect, as well as on whether there are plans for further sequencing of other isolates collected.

Reviewer #2: The manuscript has well read English . The current study uses bench top sequencer , illumina sequencing and phenotype characterization to validate the co-inhabitation of methylobacteria and NTM. It is interesting that NTMs from Hawai has antibiotic , metal resistance genes which could support their survival in Hawai. The authors have validated the MinION using Illumina and microbiological phenotype characterization authenticating their findings

Reviewer #3: The manuscript entitled “Complete Genome Assembly of Hawai’i Environmental Nontuberculous Mycobacteria Reveals Unexpected Co-Isolation with Methylobacteria” is an interesting and well written manuscript, where the authors collected thousands of environmental samples for sequencing from a Hawai’i one of the highest incidence of NTM lung disease. They also found evidence of co-occurring Methylobacteria, revealed from the sequencing data, suggesting that in some cases Methylobacteria and NTM may coexist in the same niche.

Reviewer #4: Comments:

1. Earlier studies have shown that biofilms play a very important role in preventing the co-existence of NTM and methylobacteria (PMID: 28906463, PMID: 31481764). Did the authors investigate the biofilm forming genes in the current study?

Minor comments:

1. Line no. 180, modify as ‘Gram stained as per manufacturer’s instructions’

2. Reference number 10 can be given in a proper format

**********

6. PLOS authors have the option to publish the peer review history of their article (what does this mean?). If published, this will include your full peer review and any attached files.

If you choose “no”, your identity will remain anonymous but your review may still be made public.

**Do you want your identity to be public for this peer review?** For information about this choice, including consent withdrawal, please see our Privacy Policy.

Reviewer #1: No

Reviewer #2: No

Reviewer #3: No

Reviewer #4: No

**********

[NOTE: If reviewer comments were submitted as an attachment file, they will be attached to this email and accessible via the submission site. Please log into your account, locate the manuscript record, and check for the action link "View Attachments". If this link does not appear, there are no attachment files.]

While revising your submission, please upload your figure files to the Preflight Analysis and Conversion Engine (PACE) digital diagnostic tool, https://pacev2.apexcovantage.com/. PACE helps ensure that figures meet PLOS requirements. To use PACE, you must first register as a user. Registration is free. Then, login and navigate to the UPLOAD tab, where you will find detailed instructions on how to use the tool. If you encounter any issues or have any questions when using PACE, please email PLOS at figures@plos.org. Please note that Supporting Information files do not need this step.

Attachment Submitted filename: Comments.docx Click here for additional data file.

Attachment Submitted filename: Comments for PLoSOne.doc Click here for additional data file.

# Author comment: Author response to Decision Letter 0

**DOI:** 10.1371/journal.pone.0291072.r002

16 Jun 2023

Re: Revision of “Complete Genome Assembly of Hawai’i Environmental Nontuberculous Mycobacteria Reveals Unexpected Co-Isolation with Methylobacteria.”

Dear PLOS ONE editors, 

We are grateful to the Reviewers for their insightful comments and helpful suggestions to our manuscript. We have addressed each of the Reviewers’ comments below and revised the manuscript accordingly. We are excited to potentially publish this article in PLOS ONE and thank you for your consideration.

REVIEWER #1:

Comment 1: The authors mention that thousands of isolates were collected and four were sequenced. It is not clear why the authors selected only four isolates and why these particular isolates were selected. Further detail should be given on this aspect, as well as on whether there are plans for further sequencing of other isolates collected.

Response: 

The text at lines 107-112 in the main text has been modified to include our reasoning for choosing these four particular samples: “Bacterial samples were collected from the Hawaiʻi community science project following methods previously described [6]. Four of these samples were selected for whole genome sequencing based on their unexpected pink pigmentation, which is typical of methylobacteria but not of NTM. Initial species identifications were determined through single locus PCR amplification and Sanger sequencing of the rpoB gene, which resulted in a mycobacterial identification for each sample. The samples were named HI01-04.”

Our future intention is to continue sequencing of the samples collected by the community science project and this has been added to the main text on lines 398-405: “To date, thousands of environmental samples have been collected across the Hawaiian Islands. In addition to these four samples that were sequenced by both long read and short read sequencing, we also plan to undertake short read genomic sequencing of hundreds of more NTM isolates, and to perform long read sequencing on a select number of these to produce high quality complete genome assemblies as described here. As more of these samples are fully sequenced and assembled, we aim to further elucidate the genomic diversity of Hawai’i environmental and clinical NTM, in order to better understand the high NTM infection burden in the state of Hawai’i and to shed further light on the co-existence of NTM and methylobacteria.”

REVIEWER #4:

Comment 1: Earlier studies have shown that biofilms play a very important role in preventing the co-existence of NTM and methylobacteria (PMID: 28906463, PMID: 31481764). Did the authors investigate the biofilm forming genes in the current study?

Response:

In the main document, text was added on lines 319-328 to discuss the presence of biofilm producing genes: “The finding of methylobacteria and NTM co-existence was surprising given prior reports suggesting that NTM and methylobacteria rarely occur together in the same environment [55] and never in the same biofilm [54]. A number of putative biofilm producing genes were found in the NTM genomes, including the non-ribosomal peptide synthetases pstA1, pstB1, pstC2 [56,57] and the methylobacterial genomes, including sucA [56], pstA, pstB, pstC, suggesting that all of the isolates may be capable of producing biofilms. Some literature suggests that methylobacteria interfere with NTM biofilms either by preventing adhesion to a surface [55] or degrading an existing NTM biofilm [58] but the exact compound or method remains unknown. If this is the case, it is possible that the methylobacteria in this study lacked the mechanism for preventing or degrading NTM biofilms resulting in the co-existing populations.”

Comment 2: Line no. 180, modify as ‘Gram stained as per manufacturer’s instructions’

Response: The text on line 186 was changed from “Gram stained (Fluka Analytical) per manufacturer’s instruction” to “Gram stained (Fluka Analytical) as per manufacturer’s instruction” with the addition of the word “as” in response to the reviewer’s comment.

Comment 3: Reference number 10 can be given in a proper format

Response: Additional information including website url and access data have been added to reference number 10 in the References list. Line 461 originally read “10. ‘Aina-Informatics.” and has been updated to “10. ‘Aina-Informatics. https://www.nawaiekolu.org/ainainformatics. Accessed: 2022-01-01.”

REVIEWERS #2 OR #3:

Comment 1: Hawaii being a tourist hotspot, are there any reports or whether the community scientists in this study evaluated traveller loads from tuberculosis endemic countries especially in the 4 sample areas collected in this study. Where there any tuberculosis cases reported from the households or their contacts in the places of sample collection. The authors have validated the MinION using Illumina and microbiological phenotype characterization authenticating their findings. Was co-inhabitation of methylobacteria caused any TB disease in those areas during the sample collection period or post collection if any follow-up was done.

Response: We thank the reviewer for these comments all related to tuberculosis. Respectively, we stress that this study was strictly a broad NTM environmental survey to characterize the genomic diversity of environmental NTM; thus, clinical data was not requested or collected as there was no patient participation here, tuberculosis or NTM or otherwise. 

Community scientists in this project did not evaluate traveller loads from tuberculosis endemic countries, as this was not a study aim. Since the bacteria that cause tuberculosis (M. tuberculosis – an obligate human pathogen species that cannot sustain long periods in the environment) are distinct from NTM (environmental bacteria that can reside efficiently in the environment) we believe that that the environmental NTM is not seeded by travelers, but instead that NTM infections are predominantly acquired from NTM that are already present in the environmental locations. 

We are not aware of instances where methylobacteria contributes directly to tuberculosis or NTM disease. Mycobacterial diseases are typically diagnosed by microbiologic and molecular methods that identify the presence of mycobacteria (M. tuberculosis or NTM), but not necessarily other infecting microorganisms. 

Of note, we report a change of institution for co-senior author, Dr. Jennifer Honda and have noted this update on the Facepage and where applicable.

Also of note, we added references 55-58 to the manuscript to address reviewer comments. 

Thank you again for your consideration for publication in PLOS ONE.

Sincerely,

Jo Hendrix, Ph.D.

and

Michael Strong, Ph.D.

Associate Professor

Center for Genes, Environment and Health 

National Jewish Health, Denver, CO, 80206 

StrongM@NJHealth.org , 303-270-2782

Attachment Submitted filename: Response to Reviewers.doc Click here for additional data file.

# Peer review: Decision Letter 1

**DOI:** 10.1371/journal.pone.0291072.r003

**Authors:** Divakar Sharma

11 Jul 2023

PONE-D-23-05502R1Complete genome assembly of Hawai’i environmental nontuberculous mycobacteria reveals unexpected co-isolation with methylobacteriaPLOS ONE

Dear Dr. Hendrix,

Thank you for submitting your manuscript to PLOS ONE. After careful consideration, we feel that it has merit but does not fully meet PLOS ONE’s publication criteria as it currently stands. Therefore, we invite you to submit a revised version of the manuscript that addresses the points raised during the review process.

Please submit your revised manuscript by Aug 25 2023 11:59PM.  If you will need more time than this to complete your revisions, please reply to this message or contact the journal office at plosone@plos.org. When you're ready to submit your revision, log on to https://www.editorialmanager.com/pone/ and select the 'Submissions Needing Revision' folder to locate your manuscript file.

Please include the following items when submitting your revised manuscript:

- A rebuttal letter that responds to each point raised by the academic editor and reviewer(s). You should upload this letter as a separate file labeled 'Response to Reviewers'.
- A marked-up copy of your manuscript that highlights changes made to the original version. You should upload this as a separate file labeled 'Revised Manuscript with Track Changes'.
- An unmarked version of your revised paper without tracked changes. You should upload this as a separate file labeled 'Manuscript'.

If you would like to make changes to your financial disclosure, please include your updated statement in your cover letter. Guidelines for resubmitting your figure files are available below the reviewer comments at the end of this letter.

If applicable, we recommend that you deposit your laboratory protocols in protocols.io to enhance the reproducibility of your results. Protocols.io assigns your protocol its own identifier (DOI) so that it can be cited independently in the future. For instructions see: https://journals.plos.org/plosone/s/submission-guidelines#loc-laboratory-protocols. Additionally, PLOS ONE offers an option for publishing peer-reviewed Lab Protocol articles, which describe protocols hosted on protocols.io. Read more information on sharing protocols at https://plos.org/protocols?utm_medium=editorial-email&utm_source=authorletters&utm_campaign=protocols.

We look forward to receiving your revised manuscript.

Kind regards,

Divakar Sharma

Academic Editor

PLOS ONE

Journal Requirements:

Please review your reference list to ensure that it is complete and correct. If you have cited papers that have been retracted, please include the rationale for doing so in the manuscript text, or remove these references and replace them with relevant current references. Any changes to the reference list should be mentioned in the rebuttal letter that accompanies your revised manuscript. If you need to cite a retracted article, indicate the article’s retracted status in the References list and also include a citation and full reference for the retraction notice.

**Additional Editor Comments:**

Revision requested

[Note: HTML markup is below. Please do not edit.]

Reviewers' comments:

Reviewer's Responses to Questions

**Comments to the Author**

1. If the authors have adequately addressed your comments raised in a previous round of review and you feel that this manuscript is now acceptable for publication, you may indicate that here to bypass the “Comments to the Author” section, enter your conflict of interest statement in the “Confidential to Editor” section, and submit your "Accept" recommendation.

Reviewer #1: All comments have been addressed

Reviewer #2: All comments have been addressed

Reviewer #4: All comments have been addressed

**********

2. Is the manuscript technically sound, and do the data support the conclusions?

The manuscript must describe a technically sound piece of scientific research with data that supports the conclusions. Experiments must have been conducted rigorously, with appropriate controls, replication, and sample sizes. The conclusions must be drawn appropriately based on the data presented. 

Reviewer #1: Yes

Reviewer #2: Yes

Reviewer #4: Yes

**********

3. Has the statistical analysis been performed appropriately and rigorously? 

Reviewer #1: Yes

Reviewer #2: I Don't Know

Reviewer #4: Yes

**********

4. Have the authors made all data underlying the findings in their manuscript fully available?

The PLOS Data policy requires authors to make all data underlying the findings described in their manuscript fully available without restriction, with rare exception (please refer to the Data Availability Statement in the manuscript PDF file). The data should be provided as part of the manuscript or its supporting information, or deposited to a public repository. For example, in addition to summary statistics, the data points behind means, medians and variance measures should be available. If there are restrictions on publicly sharing data—e.g. participant privacy or use of data from a third party—those must be specified.

Reviewer #1: Yes

Reviewer #2: Yes

Reviewer #4: Yes

**********

5. Is the manuscript presented in an intelligible fashion and written in standard English?

PLOS ONE does not copyedit accepted manuscripts, so the language in submitted articles must be clear, correct, and unambiguous. Any typographical or grammatical errors should be corrected at revision, so please note any specific errors here.

Reviewer #1: Yes

Reviewer #2: Yes

Reviewer #4: Yes

**********

6. Review Comments to the Author

Please use the space provided to explain your answers to the questions above. You may also include additional comments for the author, including concerns about dual publication, research ethics, or publication ethics. (Please upload your review as an attachment if it exceeds 20,000 characters)

Reviewer #1: The authors have adequately responded to all comments. The authors correctly note that seeding of M TB is completley irrelevant to this study, whose aim related to environmental mycobacteria

Reviewer #2: Dear Editor,

The author’s have provided answers to the queries raised. However, the answer to the question raised by reviewer 4 about biofilm formation remains unclear from the author’s response.

1. Please report whether biofilm formation genes were identified in the current study

2. Please report whether any biofilm formations studies were done – it seems that authors did not perform this assay. Please rephrase , Some literature suggests that methylobacteria interfere with NTM biofilms either by preventing adhesion to a surface [55] or degrading an existing NTM biofilm [58] but the exact compound or method remains unknown. If this is the case, it is possible that the methylobacteria in this study lacked the mechanism for preventing or degrading NTM biofilms resulting in the co-existing populations.” Accordingly.

Reviewer #4: The authors have adequately addressed the comments and have made necessary corrections in the manuscript.

**********

7. PLOS authors have the option to publish the peer review history of their article (what does this mean?). If published, this will include your full peer review and any attached files.

If you choose “no”, your identity will remain anonymous but your review may still be made public.

**Do you want your identity to be public for this peer review?** For information about this choice, including consent withdrawal, please see our Privacy Policy.

Reviewer #1: No

Reviewer #2: No

Reviewer #4: No

**********

[NOTE: If reviewer comments were submitted as an attachment file, they will be attached to this email and accessible via the submission site. Please log into your account, locate the manuscript record, and check for the action link "View Attachments". If this link does not appear, there are no attachment files.]

While revising your submission, please upload your figure files to the Preflight Analysis and Conversion Engine (PACE) digital diagnostic tool, https://pacev2.apexcovantage.com/. PACE helps ensure that figures meet PLOS requirements. To use PACE, you must first register as a user. Registration is free. Then, login and navigate to the UPLOAD tab, where you will find detailed instructions on how to use the tool. If you encounter any issues or have any questions when using PACE, please email PLOS at figures@plos.org. Please note that Supporting Information files do not need this step.

Attachment Submitted filename: Minor comments.docx Click here for additional data file.

# Author comment: Author response to Decision Letter 1

**DOI:** 10.1371/journal.pone.0291072.r004

7 Aug 2023

Comment 1: Please report whether biofilm formation genes were identified in the current study.

Response: 

We had added revised text regarding potential biofilm genes on lines 566-569 of the manuscript: “A number of putative biofilm producing genes were found in the NTM genomes, including the non-ribosomal peptide synthetases pstA1, pstB1, pstC2 [56,57] and the methylobacterial genomes, including sucA [56], pstA, pstB, pstC, suggesting that all of the isolates may be capable of producing biofilms.”

Comment 2: Please report whether any biofilm formations studies were done – it seems that authors did not perform this assay. Please rephrase , Some literature suggests that methylobacteria interfere with NTM biofilms either by preventing adhesion to a surface [55] or degrading an existing NTM biofilm [58] but the exact compound or method remains unknown. If this is the case, it is possible that the methylobacteria in this study lacked the mechanism for preventing or degrading NTM biofilms resulting in the co-existing populations.”

Response:

We appreciate the comment. Biofilm assays were beyond the study scope of this particular project, as this was a genomic focused study and educational experience for students who did not have the capacity or lab safety measures in place in the high school lab to address NTM biofilms. While Methylobacteria is BSL1, a biofilm assay to compare the two could not have been done by the students since NTM requires BSL2. 

In response to the reviewer’s comment, we have revised the text on line 569-574 from “Some literature suggests that methylobacteria interfere with NTM biofilms either by preventing adhesion to a surface [55] or degrading an existing NTM biofilm [58] but the exact compound or method remains unknown. If this is the case, it is possible that the methylobacteria in this study lacked the mechanism for preventing or degrading NTM biofilms resulting in the co-existing populations.” to now read “Although we did not perform biofilm experiments as part of this study, some literature suggests that methylobacteria may interfere with NTM biofilms either by preventing adhesion to a surface [55] or degrading an existing NTM biofilm [58] but the exact compound or method remains unknown. If this is the case, it is possible that the methylobacteria in this study lacked the mechanism for preventing or degrading NTM biofilms resulting in the co-existing populations.”

Attachment Submitted filename: Response to Reviewers.doc Click here for additional data file.

# Peer review: Decision Letter 2

**DOI:** 10.1371/journal.pone.0291072.r005

**Authors:** Divakar Sharma

22 Aug 2023

Complete genome assembly of Hawai’i environmental nontuberculous mycobacteria reveals unexpected co-isolation with methylobacteria

PONE-D-23-05502R2

Dear Dr. Hendrix,

We’re pleased to inform you that your manuscript has been judged scientifically suitable for publication and will be formally accepted for publication once it meets all outstanding technical requirements.

Within one week, you’ll receive an e-mail detailing the required amendments. When these have been addressed, you’ll receive a formal acceptance letter and your manuscript will be scheduled for publication.

An invoice for payment will follow shortly after the formal acceptance. To ensure an efficient process, please log into Editorial Manager at http://www.editorialmanager.com/pone/, click the 'Update My Information' link at the top of the page, and double check that your user information is up-to-date. If you have any billing related questions, please contact our Author Billing department directly at authorbilling@plos.org.

If your institution or institutions have a press office, please notify them about your upcoming paper to help maximize its impact. If they’ll be preparing press materials, please inform our press team as soon as possible -- no later than 48 hours after receiving the formal acceptance. Your manuscript will remain under strict press embargo until 2 pm Eastern Time on the date of publication. For more information, please contact onepress@plos.org.

Kind regards,

Divakar Sharma Ph.D.

Academic Editor

PLOS ONE

Additional Editor Comments (optional):

Accept

Reviewers' comments:

Reviewer's Responses to Questions

**Comments to the Author**

1. If the authors have adequately addressed your comments raised in a previous round of review and you feel that this manuscript is now acceptable for publication, you may indicate that here to bypass the “Comments to the Author” section, enter your conflict of interest statement in the “Confidential to Editor” section, and submit your "Accept" recommendation.

Reviewer #1: All comments have been addressed

**********

2. Is the manuscript technically sound, and do the data support the conclusions?

The manuscript must describe a technically sound piece of scientific research with data that supports the conclusions. Experiments must have been conducted rigorously, with appropriate controls, replication, and sample sizes. The conclusions must be drawn appropriately based on the data presented. 

Reviewer #1: (No Response)

**********

3. Has the statistical analysis been performed appropriately and rigorously? 

Reviewer #1: (No Response)

**********

4. Have the authors made all data underlying the findings in their manuscript fully available?

The PLOS Data policy requires authors to make all data underlying the findings described in their manuscript fully available without restriction, with rare exception (please refer to the Data Availability Statement in the manuscript PDF file). The data should be provided as part of the manuscript or its supporting information, or deposited to a public repository. For example, in addition to summary statistics, the data points behind means, medians and variance measures should be available. If there are restrictions on publicly sharing data—e.g. participant privacy or use of data from a third party—those must be specified.

Reviewer #1: (No Response)

**********

5. Is the manuscript presented in an intelligible fashion and written in standard English?

PLOS ONE does not copyedit accepted manuscripts, so the language in submitted articles must be clear, correct, and unambiguous. Any typographical or grammatical errors should be corrected at revision, so please note any specific errors here.

Reviewer #1: (No Response)

**********

6. Review Comments to the Author

Please use the space provided to explain your answers to the questions above. You may also include additional comments for the author, including concerns about dual publication, research ethics, or publication ethics. (Please upload your review as an attachment if it exceeds 20,000 characters)

Reviewer #1: (No Response)

**********

7. PLOS authors have the option to publish the peer review history of their article (what does this mean?). If published, this will include your full peer review and any attached files.

If you choose “no”, your identity will remain anonymous but your review may still be made public.

**Do you want your identity to be public for this peer review?** For information about this choice, including consent withdrawal, please see our Privacy Policy.

Reviewer #1: No

**********

# Editor report: Acceptance letter

**DOI:** 10.1371/journal.pone.0291072.r006

**Authors:** Divakar Sharma

1 Sep 2023

PONE-D-23-05502R2 

Complete genome assembly of Hawai’i environmental nontuberculous mycobacteria reveals unexpected co-isolation with methylobacteria 

Dear Dr. Hendrix:

I'm pleased to inform you that your manuscript has been deemed suitable for publication in PLOS ONE. Congratulations! Your manuscript is now with our production department. 

If your institution or institutions have a press office, please let them know about your upcoming paper now to help maximize its impact. If they'll be preparing press materials, please inform our press team within the next 48 hours. Your manuscript will remain under strict press embargo until 2 pm Eastern Time on the date of publication. For more information please contact onepress@plos.org.

If we can help with anything else, please email us at plosone@plos.org. 

Thank you for submitting your work to PLOS ONE and supporting open access. 

Kind regards, 

PLOS ONE Editorial Office Staff

on behalf of

Dr. Divakar Sharma 

Academic Editor

PLOS ONE
